# Engineering of 1,4‐Butanediol and Adipic Acid Metabolism in *Pseudomonas taiwanensis* for Upcycling to Aromatic Compounds

**DOI:** 10.1111/1751-7915.70205

**Published:** 2025-08-11

**Authors:** Leonie Op de Hipt, Yannic S. Ackermann, Hannah de Jong, Tino Polen, Benedikt Wynands, Nick Wierckx

**Affiliations:** ^1^ Institute of Bio‐ and Geosciences IBG‐1: Biotechnology Forschungszentrum Jülich Jülich Germany

**Keywords:** 1,4‐Butanediol, 4‐Coumarate, adipic acid, aromatics, metabolic engineering, plastic upcycling, *Pseudomonas taiwanensis*

## Abstract

The overwhelming amount of plastic produced is an unprecedented challenge for humanity due to the lack of end‐of‐life solutions for heterogeneous plastic wastes. One possibility is feedstock recycling of mixed plastics and complex polymers with subsequent biological funnelling and upcycling. Major depolymerisation products of common plastics such as polyurethanes, polyesters and polyamides include aliphatic dicarboxylic acids or diols such as adipic acid (AA) and 1,4‐butanediol (BDO), which can be metabolised by engineered *Pseudomonas putida* strains. However, the spectrum of upcycled compounds that can be produced from these monomers is still limited. Therefore, we extended the substrate spectrum of an aromatics‐overproducing *Pseudomonas taiwanensis* strain to AA and BDO. Adaptive laboratory evolution (ALE) followed by genome sequencing was used to identify and reverse engineer key growth‐enabling mutations. In this context, we observed a conflict between the dual objectives of fast growth on AA and efficient aromatics production, which materialised in the form of mutations in the ribosomal protein‐encoding gene *rpmE.* These mutations promote faster growth on AA at the cost of aromatics production. In contrast to *P. putida* KT2440, knockout of the repressor gene *psrA* regulating expression of genes involved in *β*‐oxidation had no positive effect on growth of *P. taiwanensis* on AA. Evolution for growth on BDO revealed several point mutations that affect expression of multiple oxidoreductases, with an identified key role for the dehydrogenase encoded by PVLB_10545. This dehydrogenase likely catalyses the initial oxidation of BDO, thus substituting for PedE, which is present in *P. putida* but absent in *P. taiwanensis* . Integration of *RpcTAL* into the Tn7 site enabled *de novo* production of 4‐coumarate with a yield of 14.4% ± 0.1% (Cmol/Cmol) from BDO and 11.5% ± 0.3% (Cmol/Cmol) from AA. Thereby, the potential of these *P. taiwanensis* strains for upcycling plastic hydrolysates to value‐added compounds was successfully demonstrated.

## Graphical abstract

Growth of an aromatics‐overproducing *Pseudomonas taiwanensis* on adipate and 1,4‐butanediol was engineered for bio‐upcycling. Adaptive laboratory evolution followed by genome sequencing was used to identify and reverse engineer growth‐enabling mutations. This revealed a conflict between the dual objectives of fast growth on adipate and aromatics production.

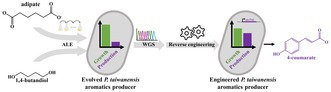

## 1 Introduction

The plastic crisis presents one of the biggest challenges of modern society. It is caused by the vast production volumes combined with the continued reliance on fossil‐based, linear systems (International Energy Agency (IEA) 2022; Geyer et al. 2017). Over 90% of plastics were still produced from virgin fossil feedstocks in 2021, while recycling rates remain low (PlasticsEurope 2022). As a result, large quantities of plastic waste accumulate in landfills and natural environments (79% as of 2015) where they persist for decades (Borrelle et al. 2020; Geyer et al. 2017; Jambeck et al. 2015; Law and Rochman 2023; Worm et al. 2017). The associated greenhouse gas emissions further contribute to the global environmental crisis (Cabernard et al. 2021; Stegmann et al. 2022). To stop this harmful and unsustainable trend, solutions leading to a more circular plastics economy are needed. In this context, bioplastics present a promising approach (Narancic et al. 2020).

One interesting candidate is the biodegradable aliphatic aromatic co‐polyester poly(butylene adipate‐*co*‐terephthalate) (PBAT), synthesised by esterifying 1,4‐butanediol (BDO) with terephthalate and successive polycondensation with adipate (AA) (Wu et al. 2023). It has similar properties to non‐biodegradable polyolefins such as LDPE, while being more accessible for biodegradation (Ferreira et al. 2017), which is why it has been introduced to the market as a more sustainable alternative more than 20 years ago and is mostly used as mulch‐foils or as packaging material in various sectors (Jian et al. 2020). However, to ensure full circularity, end‐of‐life options in the form of recycling suited for the high heterogeneity of mixed plastic wastes must be developed.

For this, a combination of chemical approaches as well as enzymatic depolymerisation might be necessary (Ellis et al. 2021; Jehanno et al. 2022; Sullivan et al. 2022). The resulting hydrolysates will be chemically diverse, making the purification of single monomers difficult and expensive. A promising and cost‐effective alternative is to use these hydrolysates as a feedstock for microbial biocatalysis (Ellis et al. 2021; Wierckx et al. 2015). The monomers are funnelled into the central metabolism of microbes and serve as a carbon and energy source for growth and, more importantly, production of desired compounds. These products, such as polyhydroxyalkanoates (PHAs) or *β*‐ketoadipic acid, can be more easily purified from the culture broth (Linger et al. 2014; Merchan et al. 2022).

Pseudomonads are promising hosts for metabolic funnelling strategies as they have a broad substrate spectrum as well as an outstanding resistance to harsh conditions such as high concentrations of plastic monomers (Bitzenhofer et al. 2021). The substrate spectrum of *Pseudomonas putida* KT2440 has already been broadened to several plastic monomers, including the PBAT monomers AA and BDO (Ackermann et al. 2021; Li et al. 2019, 2020; Qian et al. 2023). Their metabolisation has been achieved by a combination of adaptive laboratory evolution (ALE) and reverse engineering of mutations identified within the genome of the evolved strains (Ackermann et al. 2021; Li et al. 2020). Moreover, Pseudomonads have also been engineered to synthesise a variety of valuable products ranging from bioplastics to biosurfactants (Loeschcke and Thies 2015; Narancic et al. 2021; Tiso et al. 2020). One especially interesting strain is *P. taiwanensis* VLB120, which has been shown to feature a high resistance to various solvents and has already been applied to produce a wide range of aromatic compounds (Schwanemann et al. 2020). Wynands et al. (2019) have established a genome‐reduced chassis strain with enhanced flux into the shikimate pathway, which presents a superior platform strain for the production of various aromatics. For instance, production of *para*‐hydroxy aromatics such as 4‐coumarate or 4‐hydroxyphenylacetate was demonstrated (Wynands et al. 2023). These aromatic compounds have high potential to be used as building blocks for new synthetic polymers and thus can contribute to a circular plastics economy.

This is why, in this study, the substrate spectrum of a tyrosine‐overproducing *P. taiwanensis* strain is expanded to the PBAT monomers AA and BDO by ALE and reverse engineering. The aim was not only to enable the upcycling of these monomers but also to gain insights into the relationship between the dual objectives of growth and production, and how both are influenced by ALE and subsequent reverse engineering. Mutations identified in the evolved strains were largely diverging from the mutations previously identified in *P. putida* , which underlines not only the differences between the two strains but also the high potential of ALE to identify new genetic modifications for unbiased engineering. The effect of the mutations was investigated in detail, contributing to knowledge regarding the involved degradation pathways. To achieve upcycling of the PBAT monomers, a gene encoding a tyrosine ammonia‐lyase (TAL) converting tyrosine to 4‐coumarate was integrated into the *attTn7* site of the reverse engineered strains. Thereby, *de novo* production of 4‐coumarate from AA and BDO was enabled. As terephthalate, the other monomer of PBAT, is itself a valuable aromatic compound, its conversion to 4‐coumarate would not substantially enhance its value. Consequently, the aromatic production strain was not engineered for growth on terephthalate. Instead, the remaining terephthalate can be purified using a low‐energy separation process based on acid precipitation, resulting in a purity of > 95%. The recovered terephthalate can, in turn, be used for the production of new PBAT, creating a recycling loop (Ismail et al. 2024).

## 2 Results and Discussion

### 2.1 Establishing Growth on Adipate and 1,4‐Butanediol via Adaptive Laboratory Evolution

**FIGURE 1:**
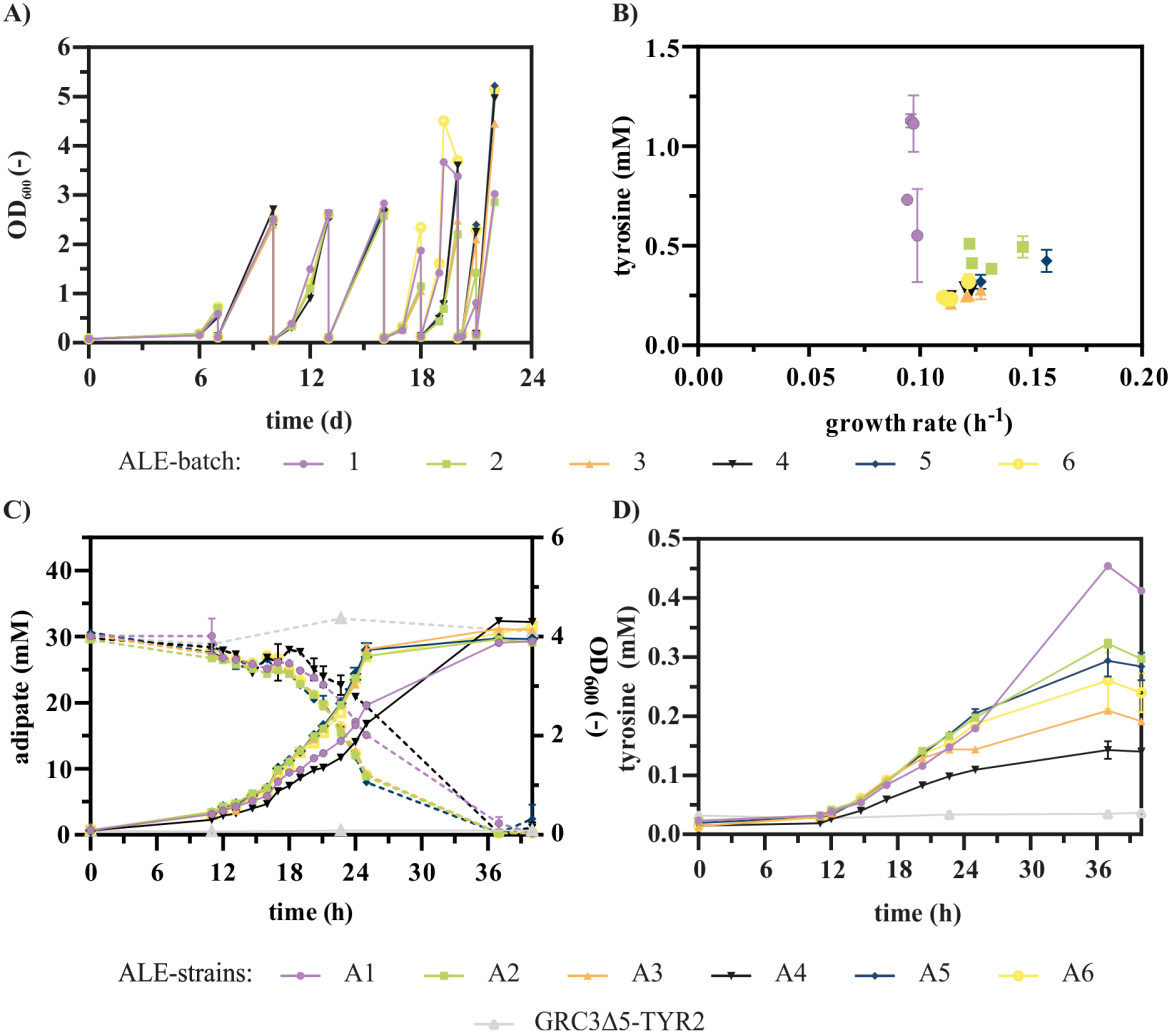
ALE of *P. taiwanensis* GRC3Δ5‐TYR2 *attTn7*::*P*_*14e*__*dcaAKIJP* on AA. (A) Sequential batch cultivation of the strain *P. taiwanensis* GRC3Δ5‐TYR2 *attTn7*::*P*_*14e*__*dcaAKIJP* in 10 mL mineral salt medium (MSM) with 30 mM AA as sole carbon source in Boston bottles. Six runs were performed in parallel. (B) Final tyrosine concentrations plotted against the growth rates of multiple clones isolated from all six ALE batches. The strains were cultivated in a Growth Profiler with online monitoring of growth via green values. To calculate the growth rates, the green values were converted into OD_600_ equivalents using a calibration. The growth rates were calculated for all cultures with the OD_600_ equivalents in the range from 0.5 to 0.9. (C) OD_600_, AA concentrations and (D) tyrosine concentrations measured at different time points during the cultivation of six selected evolved strains on 30 mM AA in shake flasks. Error bars in B were derived from three and in C and D from two replicates and indicate the standard error of the mean (SEM).

Previously, growth of *P. putida* KT2440 on AA could only be achieved with additional heterologous expression of the *dcaAKIJP* cluster from *Acinetobacter baylyi* complementing the phenylacetate degradation pathway with the enzymes enabling uptake and initial conversion of AA to the common metabolite 2,3‐didehydroadipyl‐CoA (Ackermann et al. 2021; Parke et al. 2001). Based on this knowledge, the *dcaAKIJP* was genomically integrated under control of the strong constitutive promoter *P*_*14e*_ (Zobel et al. 2015) into the *attTn7* site of *P. taiwanensis* GRC3Δ5‐TYR2, a tyrosine‐overproducing genome‐reduced chassis strain (Wynands et al. 2023). With the resulting strain, six parallel ALE runs were performed with a fixed concentration of 30 mM AA as the sole carbon source (Figure 1A). Since the aim was to allow and enhance substrate utilisation rather than tolerance, this concentration was considered sufficient.

In all runs, growth on AA was detected after a long lag phase of 6 days. After several reinoculation steps, higher OD_600_ values were achieved in shorter time periods, indicating improved growth on AA. The ALE was terminated on day 22 after approximately 32 to 37 generations, when all six cultures reached an OD_600_ above 3 within a day. While a prolonged ALE might further improve growth, it also increases the risk of accumulating random and/or adverse mutations, complicating downstream genetic analysis and aromatics production. In contrast to the evolution of *P. putida* KT2440 on AA, no additional carbon source was necessary to achieve initial growth. This is probably due to the fact that a stable and sufficient expression of the *dcaAKIJP* cluster was ensured from the beginning of the ALE via Tn7 integration (Zobel et al. 2015). In *P. putida* KT2440, a plasmid‐based expression of *dcaAKIJP* was used, which may have presented an additional bottleneck preventing growth without the application of an additional carbon source. This hypothesis is supported by mutations likely increasing the copy number of the plasmid that emerged during ALE of *P. putida* (Ackermann et al. 2021). For the selection of the strains with the most beneficial mutations, streaking of all six batches on LB agar plates was performed and single colonies were tested for growth and tyrosine production from AA (Figure 1B). From each ALE run, the strain with the best combination of growth and production was chosen for further investigation. As product formation occurs during growth, selection of strains based on a favourable combination of growth and production inherently targeted those with high overall productivity. In the following, these evolved strains will be referred to as *P. taiwanensis* A1‐6. The selected strains vary in their growth rate and tyrosine production, indicating that different mutations occurred (Figure 1C,D). Tyrosine production by the evolved strains on AA was approximately ten times lower (2.1% ± 0.0% [Cmol/Cmol]) compared to the original strain grown on 20 mM glucose (21.4% ± 0.2% [Cmol/Cmol]) (Otto et al. 2019; Wynands et al. 2023). One reason for this could be that AA is channelled into the central carbon metabolism via the TCA cycle. Therefore, the precursors for tyrosine production must be formed via gluconeogenic reactions instead of glycolysis, resulting in a higher energetic expense. A similar effect was observed before for the production of 4‐hydroxybenzoate and phenol from xylose via the TCA cycle through the oxidative Weimberg pathway (Lenzen et al. 2019; Wynands et al. 2018). Furthermore, mutations impairing the production might have occurred during the ALE, considering that aromatics production likely poses a metabolic burden, which provides a selective pressure favouring suppressor mutations.

**FIGURE 2:**
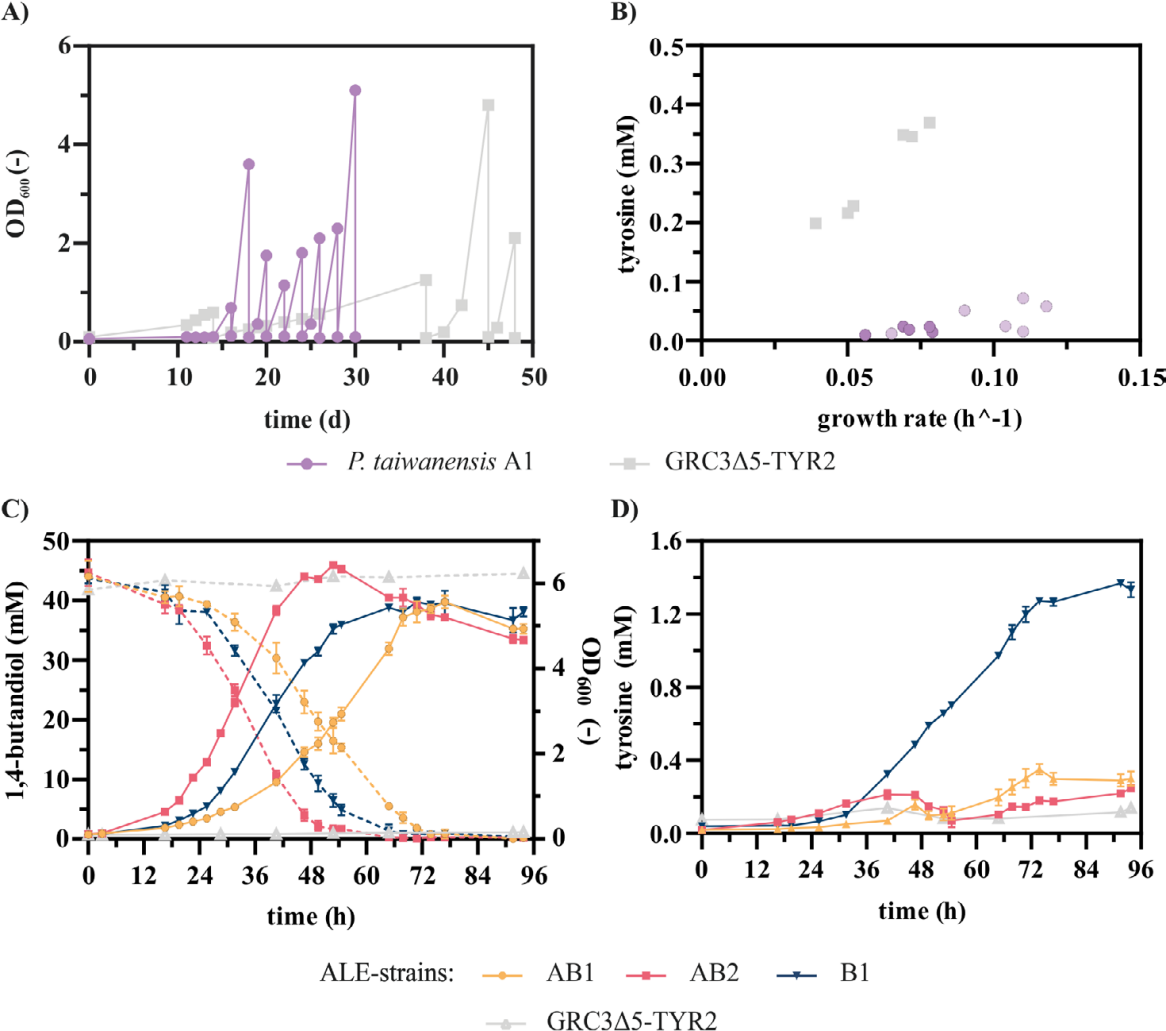
ALE of *P. taiwanensis* A1 and *P. taiwanensis* GRC3Δ5‐TYR2 on BDO. (A) Sequential batch cultivation of the strain *P. taiwanensis* A1 and *P. taiwanensis* GRC3Δ5‐TYR2 in 10 mL MSM with 45 mM BDO as sole carbon source in Boston bottles. (B) Final tyrosine concentrations plotted against growth rates of multiple strains isolated from both ALE runs. The strains were cultivated in a Growth Profiler with online monitoring of growth via green values. To calculate the growth rates, the green values were converted into OD_600_ equivalents using a calibration. The growth rates were calculated for all cultures with the OD_600_ equivalents in the range from 0.5 to 0.9. (C) OD_600_, BDO concentrations and (D) tyrosine concentrations measured at different time points during the cultivation of three selected evolved strains on 45 mM BDO in shake flasks. Two strains (AB1 and AB2) were selected from the ALE of *P. taiwanensis* A1 on BDO and one strain (B1) from the ALE of *P. taiwanensis* GRC3Δ5‐TYR2 on BDO. Error bars in C and D derive from three replicates and indicate the SEM.

In contrast to AA as carbon source, for BDO no heterologous genes are necessary to enable growth of *P. putida* KT2440 as it can be degraded via several oxidation steps catalysed by different native dehydrogenases. The gene cluster PP_2047–2051 as well as the *ped* cluster were shown to be involved in the oxidation of BDO (Li et al. 2020). However, the *ped* cluster is absent in *P. taiwanensis* VLB120 and alternative dehydrogenases oxidising BDO and intermediates needed to be identified. To that end, an ALE was carried out with the tyrosine overproducer *P. taiwanensis* GRC3Δ5‐TYR2, as well as with the abovementioned *P. taiwanensis* A1 evolved on AA (Figure 2A).

During the first 2 weeks, almost no growth on BDO was detected. Then, as before for the ALE on AA, an increase in the measured OD_600_ values and a simultaneous decrease in the required cultivation durations was observed over the course of the ALE. Single clones were isolated and tested for their growth and tyrosine production from BDO (Figure 2B). From the ALE of *P. taiwanensis* A1, which was previously evolved on AA, strains were chosen originating from two different evolutionary time points to gain more detailed insights in the improvement between different ALE steps. These strains, from now on referred to as *P. taiwanensis* AB1 and AB2, were isolated after four and nine rounds of cultivation. The respective growth rates of the isolates were 0.065 ± 0.002 h^−1^ and 0.114 ± 0.001 h^−1^ (Figure 2C). The increase in the growth rates between the two time points indicates the emergence of additional mutations. To further characterise the substrate utilisation capacity of the double‐evolved strain, cultivations were conducted using AA as sole carbon source, as well as a 1:1 mixture of AA and BDO (Figure S1). As anticipated, the strain had retained its ability to grow on AA. Interestingly, growth on the mixed substrates was markedly slower than on BDO alone and resembled the growth rate observed with AA as sole substrate. This observation indicates a potential metabolic interference or regulatory constraint when both substrates are present, reducing the growth rate.

The strain *P. taiwanensis* B1 isolated from the BDO ALE of *P. taiwanensis* GRC3Δ5‐TYR2 that was not evolved on AA before grew on BDO with a growth rate of 0.107 ± 0.002 h^−1^. Moreover, this strain produced 1.40 ± 0.02 mM tyrosine corresponding to a carbon yield of 6.9% ± 0.2% (Cmol/Cmol). This is still less than the original strain on glucose, which is likely due to the disadvantageous carbon source, but it is much more than the strains evolved on AA before, which produced tyrosine with yields in the range of 1.3% ± 0.0% (Cmol/Cmol) and 1.5% ± 0.2% (Cmol/Cmol) (Figure 2D). This supports the hypothesis that mutations occurred during the ALE on AA that impair tyrosine production.

### 2.2 Characterisation and Reverse Engineering of Causal Mutations for 1,4‐Butanediol Metabolism

**FIGURE 3:**
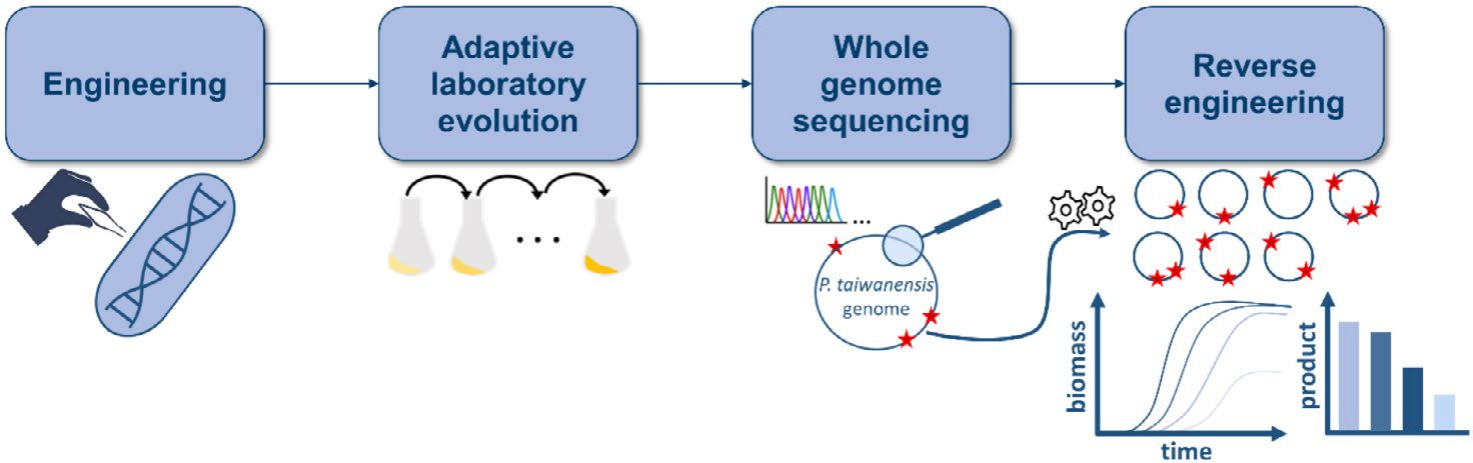
Schematic representation of the general workflow for establishing the growth of *Pseudomonas* on plastic‐derived monomers using ALE and subsequent reverse engineering. An initial engineering step is required for degradation of some plastic monomers such as AA to ensure an appropriate metabolic context for the ALE to be successful.

Adaptive laboratory evolution is a powerful approach to enable growth on non‐preferred substrates. However, the mutations are essentially random and may have unfavourable effects on the strains regarding production or growth under other conditions. Therefore, gaining a detailed knowledge of the causal mutations is essential. Hence, the genomes of selected evolved strains were sequenced and compared to the non‐evolved parental strain. Thereby, mutations are identified and can be characterised upon targeted implementation in the defined strain background of the non‐evolved strain to show their beneficial effect. This reverse engineering allows rational selection of desired mutations under consideration of potential trade‐offs, such as reduced product yields. A schematic overview of this workflow is shown in Figure 3. The most promising mutations identified within the strains evolved on BDO are shown in Table 1.

**TABLE 1:** Identified mutations in selected strains derived from the ALE on BDO.

Found in strain	Affected locus	Putative function	Mutation (position in genome)^a^	Putative effect
AB1, AB2	PVLB_10540	Small hypothetical protein	G→A (nt 2,199,154)	Silent L480L, downstream effect
AB2	PVLB_12690	LysR family transcriptional regulator	C→T (nt 2,685,347)	A247V
B1	PVLB_13305	Sigma factor dependent transcriptional regulator	T→C (nt 2,829,408)	S141P
B1	PVLB_10765	Diguanylate cyclase	C→T (nt 2,251,205)	G179D
B1	PVLB_02465 and intergenic region between PVLB_ 02465 and *putA*	Regulatory sequence, acyl‐CoA dehydrogenase	Multiple breakpoints (nt 548,394–548,999)	Alteration of coding sequence of PVLB_02465, alteration of regulatory region

^a^ Position refers to the reference genome available online at NCBI under the accession number PRJNA1111530.

**FIGURE 4:**
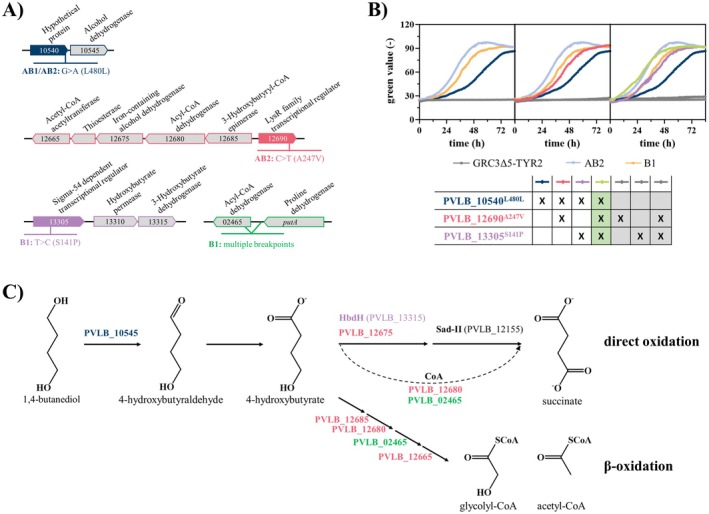
Unravelling the BDO metabolism in *P. taiwanensis* via reverse engineering. (A) Genomic context of mutations identified in the evolved strains with positive effect on growth on BDO. (B) Growth curves of engineered strains carrying different mutations found during the whole‐genome sequencing after the ALE on BDO. Non‐growing strains are marked in the table by grey background colour. All strains were cultivated in three‐fold buffered MSM containing 45 mM of BDO as sole carbon source in a Growth profiler with online monitoring of growth via green values. Error bars represent the SEM (*n* = 3). (C) Hypothetical pathway for BDO metabolism with the enzymes putatively catalysing the reactions. The colour of the enzyme corresponds to the mutations influencing the expression of encoding genes, which are schematically shown in (A). Genes encoding enzymes written in black are natively expressed.

Considering the enhanced growth rate of all selected strains on BDO as the sole carbon source, relevant mutations were expected in all three strains. The only mutation that could be identified in the strain *P. taiwanensis* AB1 was a single nucleotide variant (SNV) in PVLB_10540 encoding a hypothetical protein without known function that resulted in a silent mutation. Reverse engineering of this mutation in the non‐evolved parental strain enabled growth on BDO (Figure 4B). RT‐qPCR analysis revealed that the mutation increased expression of the downstream gene, PVLB_10545, encoding an ethanol‐active dehydrogenase (start codon 131 bp downstream of mutation) (Figure S3). Accordingly, a regulatory effect of the altered sequence can be assumed, although it could not be identified by *in silico* analysis. This downstream effect is likely responsible for the oxidation of BDO and/or its oxidation products and thereby enables its degradation. However, growth was still slower compared to the evolved strain AB2 isolated at a later ALE time point, indicating that further beneficial mutations had occurred. One additional mutation, which was present in the evolved strain AB2, was a SNV in PVLB_12690 that resulted in an amino acid exchange in a LysR family transcriptional regulator (Figure 4A). Interestingly, the homologous gene encoding a regulator with 79% amino acid sequence identity was also mutated in a *P. putida* KT2440 strain evolved on BDO (Li et al. 2020). The regulator acts as an activator of the upstream gene cluster PP_2047–51 and the mutation resulted in constitutive expression of these genes encoding an alcohol dehydrogenase and enzymes involved in *β*‐oxidation. In particular, the iron‐containing alcohol dehydrogenase encoded by PP_2049 was shown to play a role in BDO metabolisation (Li et al. 2020). In contrast to *P. putida* KT2440, reconstruction of the mutation found in PVLB_12690 alone did not enable growth on BDO (Figure 4B). This can be explained by the fact that the genome of *P. taiwanensis* lacks homologues of the *ped* cluster that was shown to be involved in BDO metabolisation in *P. putida* KT2440 (Li et al. 2020). It encodes for the two alcohol dehydrogenases PedE and PedH. Since PedH depends on lanthanides as a co‐factor that were not supplemented to the medium by Li et al. (2020) it was most likely inactive under the cultivation conditions (Mern et al. 2010; Wehrmann et al. 2017). In contrast, the calcium‐dependent alcohol dehydrogenase PedE likely catalysed the initial oxidation of BDO to 4‐hydroxybutyraldehyde. Further oxidation of the aldehyde to 4‐hydroxybutyrate was presumably catalysed by the aldehyde dehydrogenase PedI (Li et al. 2020). When combining the mutation in PVLB_12690 with the silent mutation in PVLB_10540, growth was improved over the silent mutation alone (Figure 4B). Therefore, it can be concluded that the ethanol dehydrogenase encoded by PVLB_10545 substitutes for PedE in the evolved *P. taiwanensis* strains in the initial oxidation of BDO, while the mutation in PVLB_12690 likely accelerates a limiting step downstream of 4‐hydroxybutyrate in accordance with the pathway proposed by Li et al. (2020). This hypothesis is supported by the fact that growth on 4‐hydroxybutyrate was influenced by the reconstruction of PVLB_12690^A247V^, but not by PVLB_10545^L480L^ (Figure S4). Initial slow growth followed by a rapid increase was observed, possibly caused by the intermediate accumulation of the toxic aldehyde succinate semialdehyde (Bitzenhofer et al. 2021; Jayakody et al. 2018). A similar aldehyde inhibition delay has also been observed during engineering of *P. putida* KT2440 metabolism of ethylene glycol (Franden et al. 2018). AldB‐I and PaoEFG were identified as potential substitutes for PedI, but implementation of the silent mutation in PVLB_10540 in a strain lacking AldBI and PaoEFG, *P. taiwanensis* GRC1 ROX generated by Lechtenberg et al. (2024), resulted in a strain that was able to grow on BDO. Therefore, exclusive oxidation of 4‐hydroxybutyraldehyde by these deleted dehydrogenases can be ruled out. However, a broad range of other aldehyde dehydrogenases are encoded within the *P. taiwanensis* genome that potentially could take on the role of the successive oxidation of 4‐hydroxybutyraldehyde to 4‐hydroxybutyrate.

The evolved strain *P. taiwanensis* B1 does not harbour the two mutations described above but instead one in PVLB_13305 encoding a sigma factor‐dependent transcriptional regulator and one in PVLB_10765 encoding a diguanylate cyclase, both resulting in amino acid exchanges. Additionally, a deletion of 445 bp was identified in the intergenic region between PVLB_02465 encoding an acyl‐CoA dehydrogenase and *putA* (Figure 4A). Upon reverse engineering of the first two mutations, no growth on BDO was observed (Figure 4B, Figure S5). This suggests that the growth of the evolved strain B1 is likely enabled by the deletion between PVLB_02465 and *putA*. However, our attempts to reverse engineer this mutation were unsuccessful.

A series of different combinations of mutations from both evolved strains were tested. The combined mutations in PVLB_10540, PVLB_13305, and PVLB_12690 resulted in a strain mimicking the growth phenotype of *P. taiwanensis* AB2 (Figure 4B). Hence, a positive effect of the mutation in PVLB_13305 on the growth on BDO was concluded, resulting in the fastest growth observed among the reverse engineered strains, comparable to that of the fastest evolved strain. This is probably caused by an increased expression of PVLB_13315, annotated as a 3‐hydroxybutyrate dehydrogenase, downstream of the mutation. The dehydrogenase is likely to further accelerate the oxidation of 4‐hydroxybutyrate to succinate semialdehyde, which was already identified as a limiting step by reverse engineering of PVLB_12690. In contrast, combining the silent mutation in PVLB_10540 with the mutation in the gene encoding the diguanylate cyclase (PVLB_10765) had no effect, and in combination with all three other mutations, even a negative effect was observed (Figure S5).

A suggested BDO metabolism with involved enzymes is shown in Figure 4C. In summary, initial oxidation of BDO to 4‐hydroxybutyraldehyde catalysed by PVLB_10545 and successive aldehyde oxidation by generic aldehyde dehydrogenases can be assumed. The resulting 4‐hydroxybutyrate is then further oxidised by PVLB_12675 and possibly PVLB_13315 to succinate semialdehyde, which in turn is oxidised to succinate by the succinate semialdehyde dehydrogenase Sad‐II. Additionally, oxidation via succinyl‐CoA cannot be excluded (Li et al. 2020). The acyl‐CoA dehydrogenase encoded by PVLB_12680 as well as the one encoded by PVLB_02465 upstream of the deletion are likely involved here.

As an alternative to direct oxidation of 4‐hydroxybutyrate to succinate, initial CoA activation followed by *β*‐oxidation to acetyl‐CoA and glycolyl‐CoA likely appears as well. This would be in line with the observations made by Li et al. (2020), and Ackermann et al. (2024) for *P. putida* KT2440. Moreover, this is further supported by the analysis of the evolved strains' growth on 1,6‐hexanediol (Figure S2). Growth was observed exclusively for the strain evolved on AA and BDO, indicating metabolism via AA. However, following deletion of the *dcaAKIJP* gene cluster, which is essential for AA metabolisation, minor growth was still detectable. This suggests additional metabolisation via *β*‐oxidation.

In conclusion, the growth performance of the best BDO ALE‐derived mutant AB2 was successfully reconstructed by reverse engineering. However, this was only possible through the implementation of an additional mutation (in PVLB_13305) from strain B1. This suggests that there are other effects within AB2 that positively affected growth, possibly related to the reduced tyrosine production that occurred after the primary evolution of the strain on AA. The reverse engineering resulted in a strain which grows as good as the best evolved strain while ensuring a clean genetic background without mutations potentially compromising production. The fastest growing strain resulting from the reverse engineering approach will be referred to as *P. taiwanensis* GRC3Δ5‐TYR2 BDO from here onwards.

### 2.3 Characterisation and Reverse Engineering of Causal Mutations for Adipic Acid Metabolism

**FIGURE 5:**
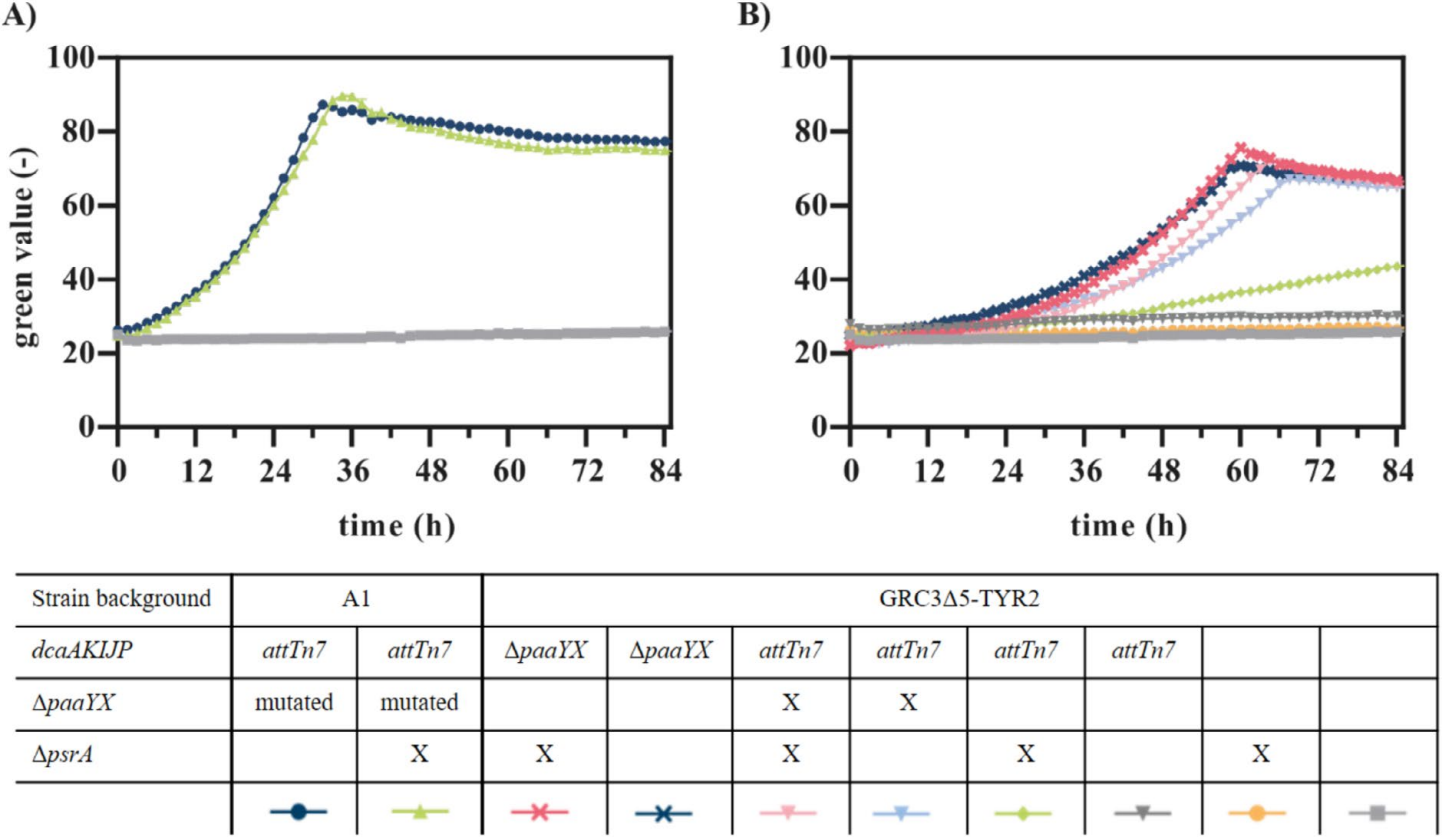
Reverse engineering of *P. taiwanensis* GRC3Δ5‐TYR2 for growth on AA. All strains were cultivated in three‐fold buffered MSM containing 30 mM of AA as sole carbon source in a Growth profiler with online monitoring of growth via green values. (A) Analysis of the growth of the evolved strain A1 on AA and of the influence of the deletion of *psrA*. (B) Growth curves of engineered strains carrying different mutations found during the whole‐genome sequencing of the ALE strains. Error bars represent the SEM (*n* = 3).

In all evolved strains isolated from the ALE on AA, mutations were identified in the genomic region of *paaXY* as well as *rpmE* (Table 2). In a previous study where *P. putida* KT2440 was evolved on AA, a transposon insertion upstream of *paaXY* led to the activation of expression of the *paa* gene cluster through the formation of a fusion promoter (Ackermann et al. 2021). Moreover, knockout of the genes encoding the PaaXY repressors combined with heterologous expression of *dcaAKIJP* enabled growth of *P. putida* KT2440 on AA as a sole carbon source, suggesting the importance of the activated phenylacetate degradation pathway. This importance was confirmed by the mutations identified around *paaX* in all six *P. taiwanensis* strains evolved on AA, including a deletion of 402 bp, SNVs in the coding sequence, as well as upstream mutations. Therefore, for reverse engineering, *paaYX* was deleted in the non‐evolved parent strain harbouring the *dcaAKIJP* genes within *attTn7*. This already resulted in growth on AA as a sole carbon source, but it was much slower than that of the ALE strain A1 (Figure 5B). Since the strain was later used for a bio‐upcycling approach and therefore required further metabolic engineering and heterologous expression of production clusters, it was advantageous to keep well‐established integration sites, such as *attTn7*. For this purpose, the *dcaAKIJP* expression cassette was integrated directly into the position of the regulatory genes *paaYX*, deleting the latter in this process. The resulting strain GRC3Δ5‐TYR2 Δ*paaYX*::*P*_*14e*_‐*dcaAKIJP* was also able to grow on AA as a sole carbon source. Furthermore, growth could be slightly increased compared to the integration at the *attTn7* site, suggesting that integration at *paaYX* is beneficial, potentially due to a higher expression rate of the *dcaAKIJP* cluster in this locus. However, growth was still not comparable to that of the evolved strains.

**TABLE 2:** Identified mutations in selected strains derived from the ALE on AA.

Found in strain	Affected locus	Putative function	Mutation (position in genome)^a^	Putative effect
A1	*paaX*	Repressor	Deletion of 2,783,326 to 2,783,539	Loss of function due to deletion and frameshift
	*rpmE*	Ribosomal protein bL31	GT→AC (469,189‐469,190)	G54D
A2	*paaX*	Repressor	T→C (2,783,610)	F209L
	*rpmE*	Ribosomal protein bL31	T→A (469,137)	C37S
A3	Intergenic region between *paaF* and *paaY*	Regulatory sequence	G→A (2,782,102)	Alteration of regulatory region, downstream effect
	Intergenic region between *priA* and *rpmE*	Regulatory sequence	T→C (468,958)	Alteration of regulatory region, downstream effect
A4	Intergenic region between *paaF* and *paaY*	Regulatory sequence	A→T (2,782,104)	Alteration of regulatory region, downstream effect
	Intergenic region between *priA* and *rpmE* and within *rpmE*	Ribosomal protein bL31	Multiple breakpoints (469,010 – 469,109)	Frameshift, alteration of coding sequence
A5	*paaX*	Repressor	C→A (2,783,700)	R239S
	Intergenic region between *priA* and *rpmE*	Regulatory sequence	A→G (468,954)	Alteration of regulatory region, downstream effect
A6	Intergenic region between *paaF* and *paaY*	Regulatory sequence	T→C (2,782,103)	Alteration of regulatory region, downstream effect
	*rpmE*	Ribosomal protein bL31	Deletion of 469,118 to 469,121	Frameshift, alteration of coding sequence

^a^ Position refers to the reference genome available online at NCBI under the accession number PRJNA1111530.

Previously, it was shown that an increased *β*‐oxidation induced by deletion of *psrA* could enhance the growth of *P. putida* KT2440 on AA as the sole carbon source (Ackermann et al. 2021). It was striking that, unlike in *P. putida* , the gene encoding the *β*‐oxidation regulator PsrA was not mutated in any of the evolved *P. taiwanensis* strains, nor were any other mutations found that might have a similar effect. To investigate this difference, *psrA* was deleted in the evolved strain GRC3Δ5‐TYR2 A1 and in the reverse engineered strain harbouring the *dcaAKIJP* within *paaYX*. Deletion of *psrA* had no or only a minor effect on growth in both strains (Figure 5A), suggesting that *β*‐oxidation does not limit growth on AA when the *paa* cluster is expressed. Deletion of *psrA* without deletion of *paaYX*, however, resulted in weak growth, similar to what was observed in *P. putida* (Ackermann et al. 2021). Thus, Δ*psrA* can enable growth on AA, but *β*‐oxidation is not the limiting step in AA degradation as long as the phenylacetate degradation pathway is active. This difference between *P. putida* and the *P. taiwanensis* strains tested here can likely be attributed to the production of aromatic compounds. The GRC3Δ5‐TYR2 strain contains many genetic modifications (Wynands et al. 2019), which reduce the growth rate of *P. taiwanensis* to the point where *β*‐oxidation catalysed by the *paa* cluster‐encoded enzymes alone is likely sufficient.

**FIGURE 6:**
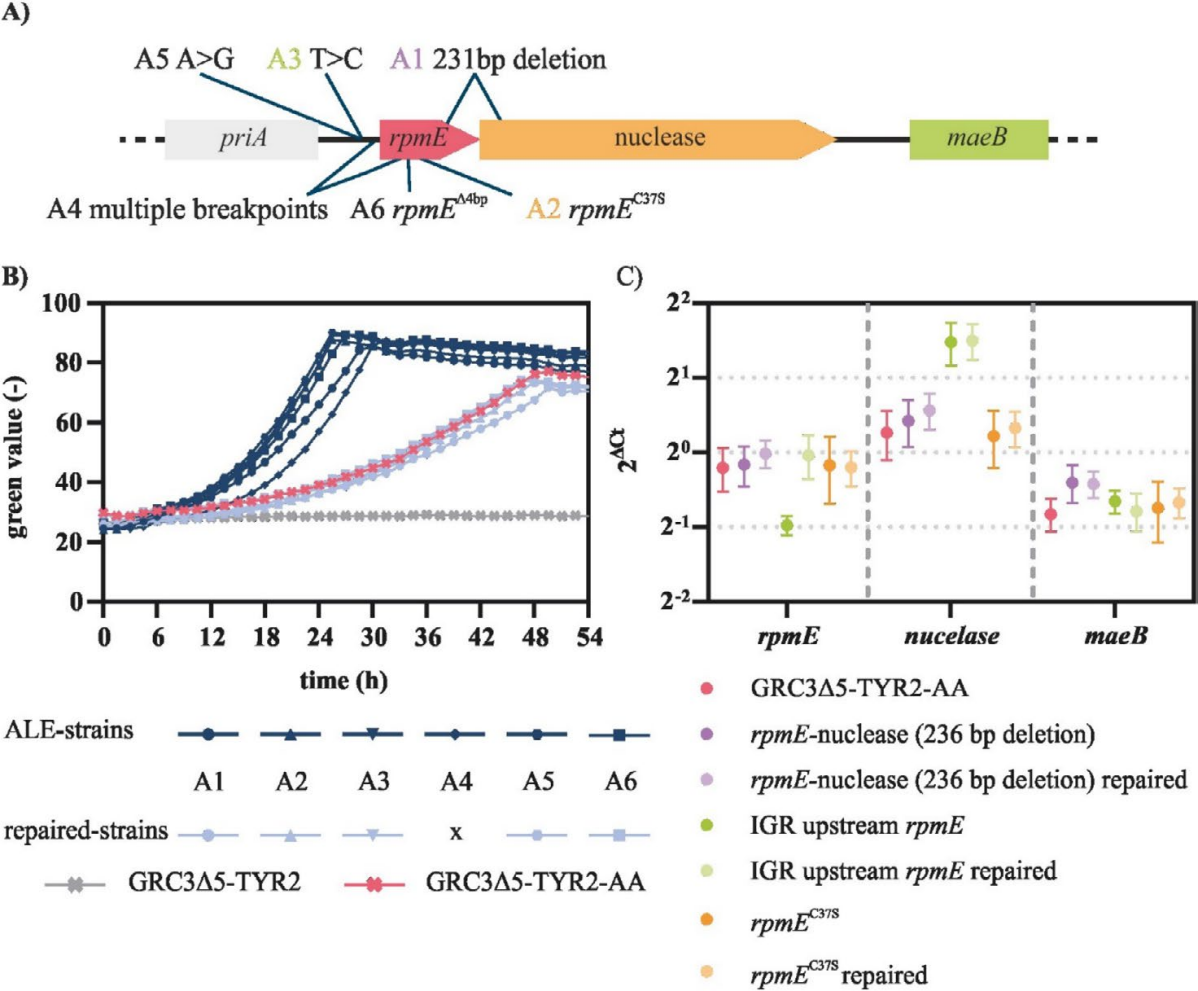
Analysis of different mutations in *rpmE* found in the ALE strains. (A) Schematic overview of the genomic region of *rpmE* and the mutations of each ALE strain. Arrow size does not correspond to gene length. (B) Comparison of growth of ALE strains with corresponding strains, which contain the wild‐type sequence of *rpmE* (repaired strains). Growth curves were measured in three‐fold buffered MSM containing 30 mM of AA in a Growth profiler with online monitoring of growth via green values. Error bars represent the SEM (*n* = 3). (C) Relative expression levels of *rpmE*, the gene encoding a nuclease, and *maeB* in the evolved strains and strains with the repaired sequence determined by RT‐qPCR. The Ct values were normalised to expression of *rpoD*. SEMs were calculated using three technical replicates of two biological replicates.

Reverse engineering of AA metabolism in GRC3Δ5‐TYR2 could not match the growth rates observed in the evolved strains, even with additional *psrA* deletion. This can likely be attributed to the mutations found in the genomic region around *rpmE* (PVLB_01635). These mutations were either a deletion of 231 bp in *rpmE* or point mutations in the intergenic region (IGR) between the upstream gene *priA* and *rpmE* or in *rpmE* itself (Figure 6A). To clarify the mutations' effects on growth on AA, all *rpmE* mutations were reverted to the wild‐type sequence in the evolved strains. This resulted in strains that grew much slower, similar to the reverse engineered strain (Figure 6B). This confirms that the mutations around *rpmE* were the reason for the growth difference between the evolved and the reverse engineered strains.

The *rpmE* gene encodes the zinc‐dependent ribosomal protein bL31, for which no Zn‐independent paralogs were identified in *P. taiwanensis* , unlike in other bacteria such as *E. coli* or *P. aeruginosa* (Hensley et al. 2012). Growth experiments with varying zinc concentrations were performed with the evolved strains as well as the evolved strains with *rpmE* mutations reverted to the wild‐type (Figure S6). The results suggest that while increased zinc availability positively affects strains with wild‐type *rpmE*, it does not fully account for the growth differences observed between these and the ALE strains, indicating that additional factors are involved.

For further insight into these factors, a RT‐qPCR was performed to analyse the expression of *rpmE* itself as well as that of adjacent genes in three of the ALE strains and their corresponding strains with repaired *rpmE* sequences. Downstream of *rpmE*, a putative nuclease and the malic enzyme MaeB are encoded. Hence, the mutations potentially also influence the expression of the malate dehydrogenase, which is part of the central metabolism. However, the analysis showed no significant difference in the expression levels of *maeB* or *priA*, also not for the strain with mutations in the IGR (Figure 6C). The same is true for *rpmE* in the strains harbouring a SNV or a deletion in the *rpmE* coding sequence itself. Only in case of the mutation in the IGR, more precisely in the putative promoter of *rpmE*, the RT‐qPCR results showed a lower expression level of *rpmE*. Since the mutations within the coding region of *rpmE* likely result in structural alterations of the bL31 protein, it appears that either a reduced amount or a modified form of bL31 is responsible for the observed differences in growth between the evolved strains and the reverse‐engineered or *rpmE*‐repaired strains.

**FIGURE 7:**
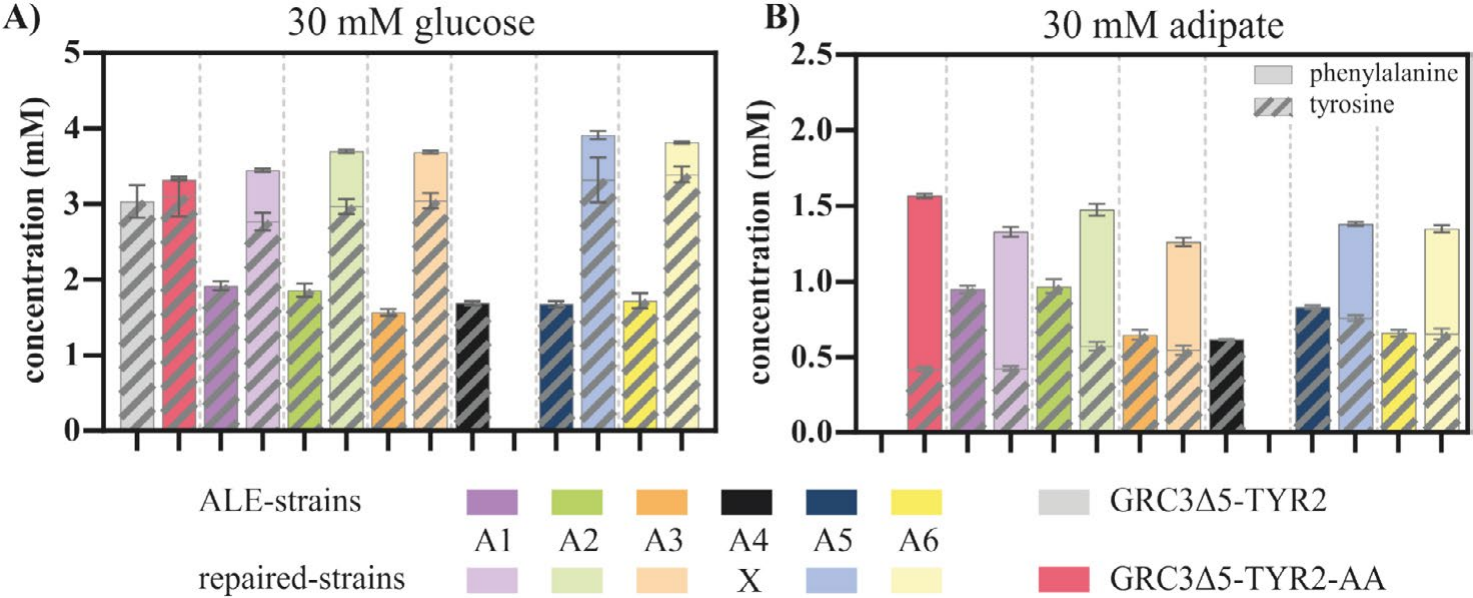
Analysis of the influence of mutated *rpmE* on tyrosine production. All strains were cultivated in three‐fold buffered MSM containing C‐equimolar concentrations of glucose and AA, and the concentrations of tyrosine and phenylalanine after 48 h of cultivation were measured via HPLC. The amounts of tyrosine and phenylalanine are added in the bar charts. Error bars represent the SEM (*n* = 3).

Considering that the parent strain is a tyrosine overproducer, tyrosine and phenylalanine concentrations after 48 h of cultivation on 30 mM glucose and 30 mM AA were measured (Figure 7). For both substrates, the evolved strains produced a significantly lower amount of aromatic amino acids (i.e., phenylalanine and tyrosine) than the strains with repaired *rpmE*, or the reverse engineered strain with wild‐type *rpmE* sequence. This confirms a link between the mutations in *rpmE* and the trade‐off between production of aromatics and growth, although it is currently not clear how the mutations in this ribosomal protein exerts this effect. The *rpmE* mutations likely affect ribosomal activity (Aseev et al. 2024), which might have an indirect global effect on intracellular amino acid pools due to altered protein synthesis rates. This could, in turn, affect tyrosine and phenylalanine production directly, or indirectly via allosteric inhibition effects in the tyrosine biosynthesis pathway. This is, however, mere speculation which requires further study. With the ultimate goal of metabolic funnelling of plastic monomers into value‐added compounds, we considered the higher yield of produced aromatics to be more important than the increase in growth rate enabled by the *rpmE* mutations, and therefore continued with a strain harbouring the *dcaAKIJP* in *paaYX* and Δ*psrA* but lacking any *rpmE* mutation. In the following, this strain is referred to as *P. taiwanensis* GRC3Δ5‐TYR2 AA.

### 2.4 4‐Coumarate Production From Adipate and 1,4‐Butanediol

For the development of an efficient bio‐upcycling approach, catabolism of monomers from plastic waste hydrolysates must be coupled to anabolism of compounds of higher value. In this study, we aimed to enable the conversion of AA and BDO into aromatic compounds by implementation of monomer metabolism in the tyrosine‐overproducing aromatics platform strain *P. taiwanensis* GRC3Δ5‐TYR2 (Wynands et al. 2019; Wynands et al. 2023). This strain is deeply engineered to increase the carbon flux through the shikimate pathway towards tyrosine (Wynands et al. 2019). 4‐Coumarate was chosen as the tyrosine‐derived target product. Besides pharma‐ and nutraceutical applications, its opposing acid and alcohol groups make it a potential building block for polymers (Timokhin et al. 2020). This makes 4‐coumarate especially interesting in the context of a plastic upcycling approach. In a previous study, Wynands et al. (2023) successfully demonstrated production of 4‐coumarate from glucose and glycerol by the genome‐reduced tyrosine platform strain also used here. However, in this work, a bottleneck was identified at the point of tyrosine deamination when tyrosine‐specific ammonia‐lyases were applied, leading to incomplete conversion of tyrosine to 4‐coumarate. This could be circumvented by the application of an unspecific *Rt*PAL accepting phenylalanine and tyrosine as substrates (PAL/TAL), but this resulted in considerable accumulation of *trans*‐cinnamate as a by‐product. A *pheA* ^P144S^ point mutation significantly decreased this by‐product formation, but at the cost of much lower growth rates.

**FIGURE 8:**
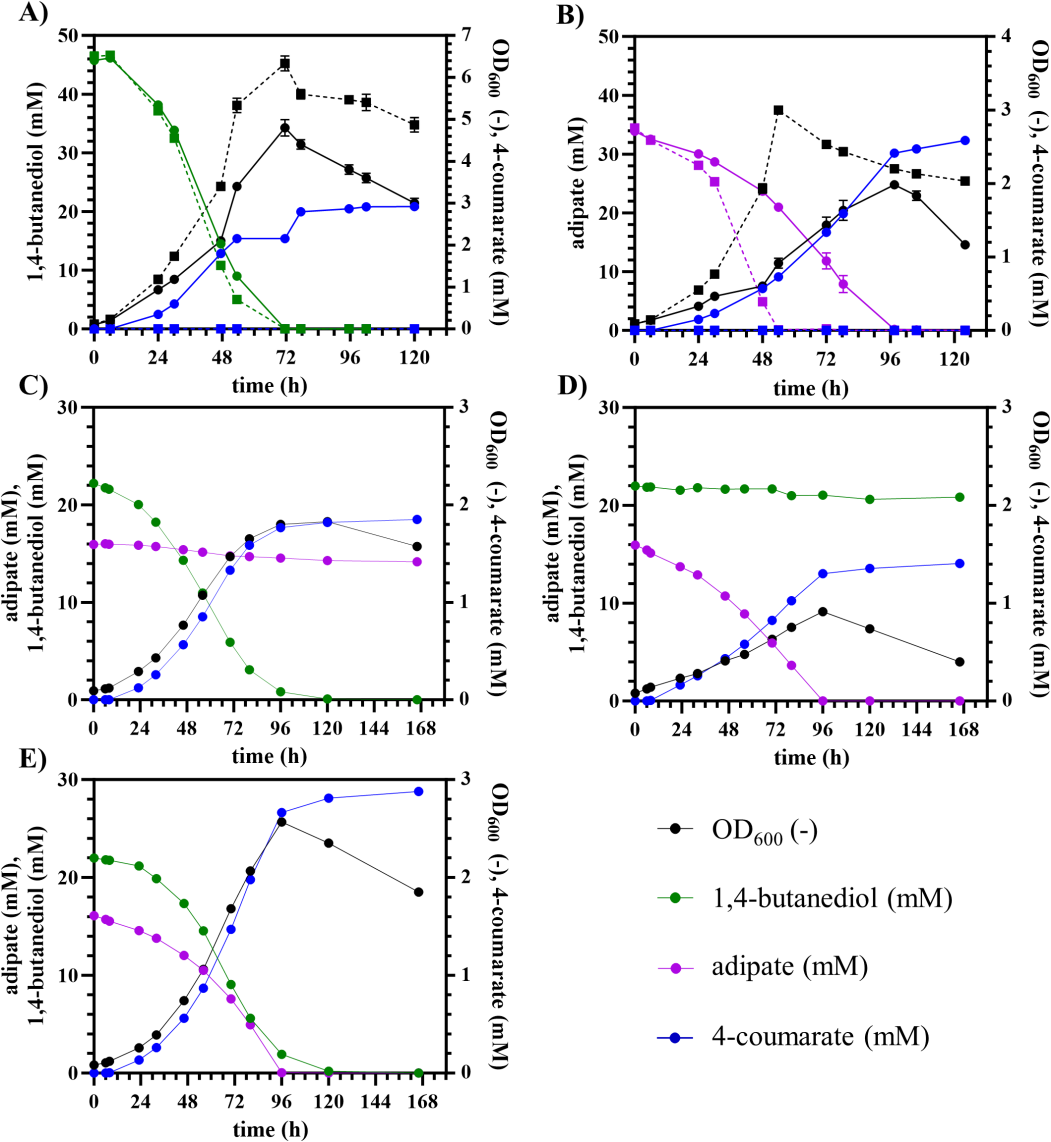
4‐Coumarate production from BDO and AA. Shake flask cultivations of the reverse engineered strains with (solid lines) and without integrated *rpcTAL* (dashed lines) on 45 mM BDO (A), 30 mM AA (B) or a mixture of 15 mM AA and 22.5 mM BDO (C,D,E). The strains were either cultivated as pure culture of the strain reverse engineered for growth on BDO (A,C) and for growth on AA (B,D) or as mixed culture of both strains (E). All cultures were inoculated with a starting OD_600_ of 0.1 (0.05 per strain in E). After 6 h, 0.1 mM salicylate was added to all cultures for induction of the *nagR/PnagAa* promoter system controlling the expression of the *rpcTAL*. Samples were taken at different time points during the cultivations, and the OD_600_ (black) as well as the concentrations of BDO (green), AA (purple) and 4‐coumarate (blue) were measured. Error bars indicate the standard error of the mean, but errors are sometimes smaller than the symbols and not visible (*n* = 3).

In this work, we therefore utilised a recently discovered *Rpc*TAL originating from the cyanobacterium *Rivularia* sp. PCC 7116, identified by Brack et al. (2022) via phylogenetic tree‐building. This *Rpc*TAL has a high specific activity of 1.45 U/mg for a tyrosine‐specific enzyme. Applying this TAL for 4‐coumarate production from glucose abolished *trans*‐cinnamate by‐product formation even without the *pheA* ^P144S^ mutation. As a consequence, producer strains grew faster and they reached a higher carbon yield of 23.9% ± 0.0% (Cmol/Cmol) compared to 19.8% ± 0.2% (Cmol/Cmol) with the *Rt*PAL combined with *pheA* ^P144S^ (Figure S7) (Wynands et al. 2023). However, integrating the same production cassette consisting of the *RpcTAL* with the strong constitutive *P*_*14f*_ promoter (Köbbing et al. 2020) in the *attTn7* site of the reverse engineered AA‐ or BDO‐metabolising strains prevented growth on the plastic monomers. The same was also true when other production modules or a degradation cluster for terephthalate were integrated into the *attTn7* site under control of the strong *P*_*14f*_ promoter (data not shown). This is likely due to high metabolic burden caused by the combination of growth on the plastic monomers accompanied by overexpression of the genes involved in the corresponding degradation pathways and the strong expression of the *RpcTAL* in the *attTn7* site (Vogeleer et al. 2024). To reduce the metabolic burden, we aimed to reduce heterologous protein expression. For this purpose, the *RpcTAL* was placed under control of the salicylate‐inducible *nagR*/*P*_*nagAa*_ promoter system allowing induction of gene expression and associated 4‐coumarate production during the growth phase. Induction after 6 h resulted in growth that was only slightly decreased compared to the non‐producing strains (Figure 8A,B). Moreover, the strains produced 4‐coumarate to concentrations of up to 3 mM from both substrates, which corresponds to carbon yields of 14.4% ± 0.1% (Cmol/Cmol) from BDO and 11.5% ± 0.3% (Cmol/Cmol) from AA (Figure 8A,B). These yields are remarkable considering that the precursors for 4‐coumarate production must be formed via reactions of gluconeogenesis, resulting in higher energetic costs (Lenzen et al. 2019; Wynands et al. 2018). An overview of the metabolic pathways involved in the conversion of AA and BDO to 4‐coumarate is provided in the supplements (Figure S8).

For further investigation of the interplay between both substrates and the influence on the conversion to 4‐coumarate, dual‐substrate cultivations were performed. The two reverse‐engineered strains harbouring *rpcTAL* were cultivated separately on a carbon‐equimolar mixture of AA and BDO (Figure 8C,D), keeping the total carbon input equivalent to that of the single‐substrate experiments (Figure 8A,B). Additionally, a mixed culture of both strains was tested under identical conditions (Figure 8E).

As expected, each strain preferentially utilises its target monomer, while the second substrate remains unconsumed. Consequently, due to the effective halving of available carbon per strain, both final OD_600_ and 4‐coumarate titers were lower than in the single‐substrate cultivations (Figure 8A,B). Nevertheless, carbon yields remained comparably high, 9.0% ± 0.0% (Cmol/Cmol) from BDO and 6.9% ± 0.0% (Cmol/Cmol) from AA, indicating that the presence of both monomers does not negatively affect substrate utilisation or product formation, a critical prerequisite for application to real hydrolysates. In the mixed culture, simultaneous and complete consumption of both monomers was observed, leading to an increased 4‐coumarate titre. The resulting carbon yield of 14.0% ± 0.1% (Cmol/Cmol) reflects the combined metabolic capacity of both strains and falls within the range of yields obtained in the single‐substrate cultivations (Figure 8A,B).

The production of the valuable compound 4‐coumarate from the plastic monomers AA and BDO demonstrates the high potential of the engineered *P. taiwanensis* strains for the upcycling of plastic waste into valuable building blocks that can be used, for example, for the production of new bio‐based polymers.

## 3 Conclusion

In this study, we extended the substrate spectrum of a tyrosine‐overproducing *P. taiwanensis* strain to AA and BDO, enabling microbial upcycling of these PBAT monomers. The ALE enabled fast and unbiased optimisation of plastic monomers metabolism, providing clear leads for rational strain engineering and highlighting differences between *P. putida* and *P. taiwanensis* . However, this work also exemplified a drawback of ALE‐based optimisation for substrate metabolism in the presence of a secondary objective of product formation. This highlights the importance of rational reverse engineering, which in the case of AA conversion restored lost production capacity. The *P. taiwanensis* GRC3∆5‐TYR2 platform can readily be extended with other production modules, enabling the bioconversion of AA and BDO into a wide range of value‐added aromatics (Schwanemann et al. 2020; Wynands et al. 2018). Here, we demonstrated the production of the *para*‐hydroxylated aromatic compound 4‐coumarate, which has possible applications in formation of new bio‐based polymers, and can also be further converted to value‐added compounds such as 4‐vinylphenol, 4‐hydroxyphenylacetate, and 4‐hydroxybenzoate (Wynands et al. 2023). This shows the potential of this platform for plastic hydrolysate upcycling, which can be further extended by implementation of other monomer‐metabolism pathways.

## 4 Experimental Procedures

### 4.1 Strains and Culture Conditions

The chemicals used in this work were obtained from Carl Roth (Karlsruhe, Germany), Sigma‐Aldrich (St. Louis, MO, USA), or Merck (Darmstadt, Germany) unless stated otherwise. All *P. taiwanensis* strains explicitly mentioned within the manuscript are listed within the following (Table 3). All other strains used in the preparation of this paper are listed in an additional Table S1 in the supplements.

**TABLE 3:** All *P. taiwanensis* strains mentioned in this study.

Strain	Description	References
GRC1 ROX	Genome‐reduced chassis of *P. taiwanensis* VLB120 with ∆pSTY, ∆prophage1/2, ∆prophage3, ∆prophage4, ∆flag1, ∆flag2, ∆lap1, ∆lap2, ∆lap3, ∆*paoE*, ∆*paoF*, ∆*paoG*, ∆*aldB‐I*	Lechtenberg et al. (2024)
GRC3∆5‐TYR2	Genome‐reduced chassis strain of *P. taiwanensis* VLB120 with ΔpSTY, Δprophage1/2::*ttgVWGHI*, Δprophage3, Δprophage4, Δflag1, Δflag2, Δlap1, Δlap2, Δlap3, Δ*pobA*, Δ*hpd*, Δ*quiC*, Δ*quiC1*, Δ*quiC2*, *trpE* ^P290S^, *aroF‐1* ^P148L^, *pheA* ^T310^, Δ*pykA*	Wynands et al. (2023)
AB1	GRC3∆5‐TYR2 A1 evolved on BDO as a sole carbon source	This work MiCat#1211
AB2	GRC3∆5‐TYR2 A1 evolved on BDO as a sole carbon source	This work MiCat#2507
B1	GRC3∆5‐TYR2 evolved on BDO as a sole carbon source	This work MiCat#1478
AB1 Δ*attTn7*::P_14e__*dcaAKIJP*	restored *attTn7* site by knockout of *dcaAKIJP*	This work MiCat#1148
GRC3∆5‐TYR2 PVLB_10540^L480L^	Reconstruction of SNV in PVLB_10540	This work MiCat#1508
GRC3∆5‐TYR2 PVLB_12690^A247V^	Reconstruction of SNV in PVLB_12690	This work MiCat#1481
GRC3∆5‐TYR2 PVLB_13305^S141P^	Reconstruction of SNV in PVLB_13305	This work MiCat#1480
GRC3∆5‐TYR2 PVLB_10540^L480L^ PVLB_12690^A247V^	Combination of SNV in PVLB_10540 and PVLB_12690	This work MiCat#1530
GRC3∆5‐TYR2 PVLB_10540^L480L^ PVLB_13305^S141P^	Combination of SNV in PVLB_10540 and PVLB_13305	This work MiCat#1509
GRC3∆5‐TYR2 PVLB_12690^A247V^ PVLB_13305^S141P^	Combination of SNV in PVLB_12690 and PVLB_13305	This work MiCat#1482
GRC3∆5‐TYR2 BDO	Combination of SNV in PVLB_10540, PVLB_12690 and PVLB_13305	This work MiCat#1486
GRC3∆5‐TYR2 *attTn7*::P_*14e*_‐*dcaAKIJP*	Genome‐integrated *dcaAKIJP* cluster under the control of P_*14e*_	This work MiCat#623
A1 – A6	GRC3∆5‐TYR2 *attTn7*::P_*14e*_‐*dcaAKIJP* evolved on AA as sole carbon source	This work MiCat#1323—MiCat#1328
GRC3∆5‐TYR2 Δ*psrA*	Knockout of *psrA*	This work MiCat#1426
A1 Δ*psrA*	Knockout of *psrA* in evolved strain A1	This work MiCat#1428
GRC3∆5‐TYR2 *attTn7*::P_*14e*_‐*dcaAKIJP* Δ*psrA*	Knockout of *psrA* in addition to genomic integration of *dcaAKIJP*	This work MiCat#1427
GRC3∆5‐TYR2 *attTn7*::P_*14e*_‐*dcaAKIJP* Δ*paaYX* Δ*psrA*	Knockout of *paaYX* and *psrA* in addition to genomic integration of *dcaAKIJP*	This work MiCat#1443
GRC3∆5‐TYR2 *attTn7*::P_*14e*_‐*dcaAKIJP* Δ*paaYX*	Knockout of *paaYX* in addition to genomic integration of *dcaAKIJP*	This work MiCat#2399
GRC3∆5‐TYR2 Δ*paaYX*:: P_*14e*_‐*dcaAKIJP*	Integration of *dcaAKIJP* at the position of *paaYX* by deleting of *paaYX*	This work MiCat#1978
GRC3∆5‐TYR2 AA	Iintegration of *dcaAKIJP* at the position of *paaYX* by deleting *paaYX*, knockout of *psrA*	This work MiCat#1434
A1 *rpmE* (repaired) – A6 *rpmE* (repaired) (except A4)	GRC3∆5‐TYR2 *attTn7*::P_*14e*_‐*dcaAKIJP* evolved on AA as sole carbon source with repaired *rpmE* region with wild‐type sequence	This work MiCat#2233‐ MiCat#2245
GRC3∆5‐TYR2 BDO *attTn7::nagR/PnagAa‐rpcTAL*	Combination of SNV in PVLB_10540, PVLB_12690 and PVLB_13305, *attTn7::Kan_FRT_nagR/PnagAa‐rpcTAL*	This work MiCat#2589
GRC3∆5‐TYR2 AA *attTn7::nagR/PnagAa‐rpcTAL*	Combined integration of *dcaAKIJP* at the position of *paaYX* by deleting *paaYX*, knockout of *psrA*, *attTn7::Kan_FRT_nagR/PnagAa‐rpcTAL*	This work MiCat#2587

All strains were routinely cultured in lysogeny broth (LB) medium prepared with premixed LB medium (Carl Roth, Karlsruhe, Germany) or on LB agar plates prepared with a respective mixture (Carl Roth, Karlsruhe, Germany). To select for *Pseudomonas* strains after mating procedures, 25 mg L^−1^ irgasan was added. For the selection of delivered genetic elements, antibiotics corresponding to the transferred resistance genes were added in the following concentrations: 50 mg L^−1^ kanamycin sulfate, 25 mg L^−1^ gentamicin, 100 mg L^−1^ ampicillin. Experiments involving the measurement of growth on plastic monomers, their utilisation, and the production of aromatic compounds were conducted using a mineral salt medium (MSM) according to Hartmans et al. (1989) with an adapted standard phosphate buffer capacity of 22.3 mM K_2_HPO_4_ and 13.6 mM NaH_2_PO_4_. Pre‐cultures were performed in MSM with 20 mM of glucose, whereas the main cultures were performed with a three‐fold buffer unless stated otherwise and the plastic monomers instead of glucose. All plastic monomers were added in a C‐equimolar concentration to 45 mM BDO. In general, liquid cultures were performed in 500 mL non‐baffled Erlenmeyer flasks with a filling volume of 10%, at 200 rpm shaking speed with an amplitude of 50 mm and a humidity of 80% using an ISF1‐X Climo‐Shaker (Kuhner shaker, Birsfelden, Switzerland). All *Pseudomonas* strains were cultivated at 30°C and all *E. coli* strains at 37°C. For online monitoring of growth, liquid cultivations were performed in a 96‐microwell plate with a transparent polystyrene bottom (Enzyscreen, Heemstedem, The Netherlands) filled with 200 μL of cell suspensions within the Growth Profiler (Enzyscreen, Heemstedem, The Netherlands) at 225 rpm with an amplitude of 50 mm and 30°C.

### 4.2 Adaptive Laboratory Evolution

ALE was performed in 100 mL Boston bottles filled with 10 mL MSM. The first ALE step was inoculated with an overnight culture grown on glucose. As soon as the cultures were turbid, they were sampled and OD_600_ was measured. If OD_600_ was above 1, fresh medium was inoculated for the next ALE step. Starting OD_600_ of every ALE step was 0.1. Cells used for inoculation were washed with sterile 0.9% (w/v) sodium chloride solution to prevent transfer of media components and allow cultivation under constant conditions. To obtain evolved strains, single colonies were isolated on LB agar plates and subsequently analysed for their growth and tyrosine production from the plastic monomers. For this, they were grown in the Growth Profiler (Enzyscreen, Heemstedem, Netherlands). Depending on growth and tyrosine production, at least one strain from each ALE batch was selected for whole‐genome sequencing and further analysis in shake flask cultivations.

### 4.3 Plasmid Cloning and Strain Engineering

All cloning primers and plasmids utilised in this work are listed in Tables S2 and S3. The primers were ordered from Eurofins Genomics (Ebersberg, Germany) and used in combination with Q5 High‐Fidelity Polymerase (New‐England Biolabs, Ipswich, MA, USA) to amplify DNA fragments via PCR. The fragments were assembled to plasmids by Gibson assembly (Gibson et al. 2009) using the NEBuilder HiFi DNA Assembly Master Mix (New‐England Biolabs, Ipswich, MA, USA). Constructed plasmids were transformed into competent *E. coli* cells via heat shock. Subsequently, the plasmids were transferred from *E. coli* into the desired *Pseudomonas* strain through conjugational transfer using *E. coli* HB101 pRK2013 as helper strain as described by Wynands et al. (2018). For genomic integration of heterologous genes into the *attTn7* site, a pBG mini‐Tn7 vector harbouring the genetic module of interest as well as the pTNS1 providing the required transposase were introduced (Zobel et al. 2015). A variant of the pBG plasmid containing the constitutive promoter *P14e* or *P14f* or the salicylate‐inducible *nagR*/*P*_*nagAa*_ promoter system as well as FRT sites for a recyclable kanamycin marker was used as described by Ackermann et al. (2021). SNVs and knockouts were obtained using the pEMG system described by Martínez‐García and de Lorenzo (2011) with a modified protocol described by Wynands et al. (2018). All constructed plasmids and genomic modifications were verified via colony PCR using One*Taq* 2X Master Mix (New England BioLabs, Ipswich, Massachusetts, USA) followed by Sanger sequencing performed by Eurofins Genomics (Ebersberg, Germany). For this purpose, template colonies were pre‐lysed with alkaline PEG 200, according to Chomczynski and Rymaszewski (2006).

### 4.4 RT‐qPCR

Gene expression levels were analysed by RT‐qPCR. For this purpose, 50 mL shake flask main cultures were carried out as previously described. After reaching the mid‐exponential growth phase, cells were harvested and promptly suspended in 1 mL of RNAlater (Thermo Fisher Scientific, Massachusetts, USA). The samples were then stored at −20°C until analysis. RNA extraction was performed using the Quick‐RNA Miniprep Kit (Zymo Research, Irvine, CA, USA) and cDNA was prepared from the purified RNA using the LunaScript RT superMix Kit (New England Biolabs, Ipswich, MA, USA). The expression levels of the housekeeping gene *rpoD* (Wang and Nomura 2010) as well as the gene of interest were analysed using oligonucleotides listed in Table S3 that were designed by the qPCR assay design tool from Eurofins Genomics. Quantitative RT‐PCR was performed using Luna Universal qPCR Master Mix (New England Biolabs, Ipswich, MA, USA) in 96‐well plates by the qTOWER 2.2 (Analytik Jena, Jena, Germany). The reaction conditions were used as described in the manufacturer's instructions. Experiments were performed in technical triplicates with biological duplicates or triplicates. Gene expression levels were evaluated by comparing the Ct values of the housekeeping gene *rpoD* (Wang and Nomura 2010) with the Ct value of genes of interest using the following equation:

$$\text{Gene expression level}=2^{Ct\left(\text{rpoD}\right)–Ct\left(\text{target}\right)}$$

### 4.5 Analytical Methods

For shake flask cultivations, growth was detected by measuring the optical density at 600 nm (OD_600_) with an Ultrospec 10 Cell Density Meter (GE Healthcare, Little Chalfront, Buckinghamshire, United Kingdom). Online growth monitoring was performed in the growth profiler via bottom‐up images of transparent‐bottom microtiter plates taken every 30 min and image analysis using the Growth Profiler Control software V2_0_0. Where indicated, resulting green values were converted to an equivalent OD_600_ via a non‐linear correlation.

### 4.6 HPLC Analysis

Concentrations of extracellular metabolites were detected via HPLC analysis. For this purpose, samples were prepared by centrifugation for 3 min at 17,000 × g and filtration of the supernatant through an AcroPrep 96‐well filter plate (Pall Corporation, Port Washington, NY, USA). All measurements were performed with the 1260 Infinity II HPLC (Agilent, Santa Clara, California, USA). For analysis of glucose, AA and BDO concentrations, the column Metab‐AAC (300 × 7.8 mm, ISERA; P.N.: A1BF‐A1AA0N) or Metab‐AAC (150 × 7.8 mm, ISERA; P.N.: A1BF‐A1A40N), both equipped with a Guard Cartridge Holder (ISERA, P.N.: AA13‐000005) and Guard Column (10 × 7.8 mm, ISERA, P.N.: A1BF‐A1AG0N) were used with 5 mM H_2_SO_4_ as mobile phase at 0.6 mL min^−1^ and column temperature of 40°C. All three compounds were detected with a 260 Infinity II Refractive Index Detector (Agilent, Santa Clara, California, USA). Tyrosine and phenylalanine were detected with a method involving the derivatization of the amino group with *ortho*‐phthalaldehyde via the 1260 Infinity II Fluorescence Detector (Agilent, Santa Clara, California, USA). For this method, the utilised column was Phenomenex Kinetex 2.6 μm EVO C18 100 Å (Phenomenex, Torrance, California, United States of America) with methanol and borate buffer (14.2 g L^−1^ Na_2_HPO_4_, 28.1 g L^−1^ Na_2_B_4_O_7_, pH at 8.2) as running agents. A constant flow of 0.42 mL min^−1^ and a gradient from 95% borate buffer and 5% methanol to 100% methanol during the first 10 min and back to the starting ratio during the last 2 min was applied. The column temperature was 40°C. For the detection of 4‐coumarate, a reversed‐phase HPLC column, InfinityLab Poroshell 120 EC‐C18 (3.0 × 150 mm, 2.7 μm, Agilent Technologies, P.N.: 693975‐302T) with guard column (Agilent Technologies; P.N.: 823750‐911) was utilised. A gradient with 0.1% (v/v) trifluoroacetic acid (Sigma‐Aldrich, Munich, Germany) and acetonitrile (Th. Geyer, Renningen, Germany) at a flow rate of 0.8 mL min^−1^ and a temperature of 40°C was applied and 4‐coumarate was UV detected and analysed at a wavelength of 240 nm with the 1260 DAD WR detector (Agilent Technologies).

### 4.7 Genome Sequencing

For whole‐genome sequencing, genomic DNA was isolated using the Monarch Genomic DNA Purification Kit (New‐England Biolabs, Ipswich, MA, USA) following the manufacturer's instructions. For library preparation, the NEBNext Ultra II DNA Library Prep Kit for Illumina (New England Biolabs, Ipswich, Massachusetts, USA) was utilised. All steps were performed according to the manufacturer's protocol. For validation of the library, qPCR was carried out with the KAPA PROBE FORCE Kit (Sigma‐Aldrich, Munich, Germany). Subsequently, the concentrations of the library samples were calculated. Samples were diluted to the desired concentration of 3 nM with 10 mM Tris/HCl containing 0.1% Tween (pH of 8.5). Denaturation of the DNA was achieved by mixing 5 μL of the library sample with 5 μL 0.2 N NaOH, short vortexing, centrifugation, and incubation for 5 min at room temperature. After incubation, 990 μL of cold hybridisation buffer HT1 from the MiSeq reagent kit (Illumina, San Diego, California, USA) used for sequencing was added. 600 μL of the prepared samples were loaded onto the prefilled cartridge and sequencing was started in the MiSeq System (Illumina, San Diego, California, USA). After the sequencing run, *de novo* assembly of the resulting sequencing reads followed by alignment to the reference genome was carried out.

Sequencing data are deposited in the NCBI Sequence Read Archive under BioProject number PRJNA1111530.

## Author Contributions

**Leonie Op de Hipt:** methodology, investigation, validation, formal analysis, data curation, writing – original draft, writing – review and editing, visualization. **Yannic S. Ackermann:** methodology, investigation, validation, formal analysis, data curation, writing – original draft, writing – review and editing, visualization. **Hannah de Jong:** investigation, writing – review and editing. **Tino Polen:** methodology, writing – review and editing, data curation, formal analysis. **Benedikt Wynands:** methodology, writing – review and editing, supervision. **Nick Wierckx:** conceptualization, resources, data curation, writing – original draft, writing – review and editing, visualization, supervision, project administration, funding acquisition.

## Conflicts of Interest

The authors declare no conflicts of interest.

## Supporting information


**Data S1:** mbt270205‐sup‐0001‐Supinfo.docx.

## Data Availability

The data that support the findings of this study are available from the corresponding author upon reasonable request.
