# Supplementary material for: Engineering of 1,4‐Butanediol and Adipic Acid Metabolism in Pseudomonas taiwanensis for Upcycling to Aromatic Compounds
Source: Microb Biotechnol. 2025 Aug 11;18(8):e70205. doi: 10.1111/1751-7915.70205 (PMC12337976; doi:10.1111/1751-7915.70205)
Supplement: Supplementary file 1 — Data S1: mbt270205‐sup‐0001‐Supinfo.docx. [file MBT2-18-e70205-s001.docx]

**Supplementary data to:**

**Engineering of 1,4-butanediol and adipic acid metabolism in *Pseudomoas taiwanensis* for upcycling to aromatic compounds**

Leonie Op de Hipt^1§^, Yannic S. Ackermann^1§^, Hannah de Jong^1^, Tino Polen^1^, Benedikt Wynands^1^, Nick Wierckx^1*^

^1^Institute of Bio- and Geosciences IBG-1: Biotechnology, Forschungszentrum Jülich, Jülich, Germany

§these authors contributed equally to this study.

*Corresponding author:
Nick Wierckx
E-mail: [n.wierckx@fz-juelich.de](mailto:n.wierckx@fz-juelich.de)

**Supplementary Information**

**Table S1: Strains used in this study.**

| Strain | Description | Reference |
| --- | --- | --- |
| *Escherichia coli* | | |
| DH5α | F􀀀 Φ80 lacZΔM15 Δ(lacZYAargF) U169 recA1 endA1 hsdR17 (rk􀀀, mk+) phoA supE44 thi-1 gyrA96 relA1 λ􀀀 | Thermo Fischer Scientific |
| DH5α λ*pir* | λ*pir* lysogen of DH5α; host for *oriV(R6K)* plasmids | Platt et al., 2000 |
| PIR2 | F􀀀 Δlac169 rpoS(Am) robA1creC510 hsdR514 endA recA1uidA(ΔMluI):pir; host for oriV (R6K) plasmids | Thermo Fischer Scientific |
| HB101 pRK2013 | HB101 with pRK2013 | Ditta et al., 1980 |
| DH5α λ*pir* pTNS1 | DH5α λ*pir*with pTNS1 | Choi et al., 2005 |
| DH5α pSW-2 | DH5α with pSW-2 | Martínez-García and de Lorenzo, 2011 |
| *Pseudomonas taiwanensis* | | |
| VLB120 | wild-type strain | Panke et al., 1998 |
| GRC1 ROX | genome-reduced chassis of *P. taiwanensis* VLB120 with ∆pSTY, ∆prophage1/2, ∆prophage3, ∆prophage4,  ∆flag1, ∆flag2, ∆lap1, ∆lap2, ∆lap3, ∆*paoE*, ∆*paoF*, ∆*paoG* , ∆*aldB-I* | Lechtenberg et al., 2024 |
| GRC3∆5-TYR2 | genome-reduced chassis strain of  *P. taiwanensis* VLB120 with ΔpSTY, Δprophage1/2::*ttgVWGHI*, Δprophage3, Δprophage4, Δflag1, Δflag2, Δlap1, Δlap2, Δlap3, Δ*pobA*, Δ*hpd*, Δ*quiC*, Δ*quiC1*, Δ*quiC2*, *trpE*^P290S^, *aroF-1*^P148L^, *pheA*^T310^, Δ*pykA*, | Wynands et al., 2023 |
| AB1 | GRC3∆5-TYR2 A1 evolved on BDO as sole carbon source | this work MiCat#1211 |
| AB2 | GRC3∆5-TYR2 A1 evolved on BDO as sole carbon source | this work MiCat#2507 |
| B1 | GRC3∆5-TYR2 evolved on BDO as sole carbon source | this work  MiCat#1478 |
| AB1 Δ*attTn7*::P_14e__*dcaAKIJP* | restored *attTn7* site by knockout of *dcaAKIJP* | this work  MiCat#1148 |
| GRC3∆5-TYR2 PVLB_10540^L480L^ | reconstruction of SNV in PVLB_10540 | this work  MiCat#1508 |
| GRC3∆5-TYR2 PVLB_12690^A247V^ | reconstruction of SNV in PVLB_12690 | this work  MiCat#1481 |
| GRC3∆5-TYR2 PVLB_13305^S141P^ | reconstruction of SNV in PVLB_13305 | this work  MiCat#1480 |
| GRC3∆5-TYR2 PVLB_10765^G179D^ | reconstruction of SNV in PVLB_10765 | this work  MiCat#1576 |
| GRC3∆5-TYR2 PVLB_10540^L480L^ PVLB_12690^A247V^ | combination of SNV in PVLB_10540 and PVLB_12690 | this work  MiCat#1530 |
| GRC3∆5-TYR2 PVLB_10540^L480L^ PVLB_13305^S141P^ | combination of SNV in PVLB_10540 and PVLB_13305 | this work  MiCat#1509 |
| GRC3∆5-TYR2 PVLB_12690^A247V^ PVLB_13305^S141P^ | combination of SNV in PVLB_12690 and PVLB_13305 | this work  MiCat#1482 |
| GRC3∆5-TYR2 PVLB_12690^A247V^ PVLB_13305^S141P^ PVLB_10765^G179D^ | combination of SNV in PVLB_12690, PVLB_13305 and PVLB_10765 | this work  MiCat#1579 |
| GRC3∆5-TYR2 BDO | combination of SNV in PVLB_10540, PVLB_12690 and PVLB_13305 | this work  MiCat#1486 |
| GRC3∆5-TYR2 PVLB_10540^L480L^ PVLB_12690^A247V^ PVLB_13305^S141P^ PVLB_10765^G179D^ | combination of SNV in PVLB_10540, PVLB_12690, PVLB_13305 and PVLB_10765 | this work  MiCat#1580 |
| GRC3∆5-TYR2 *attTn7*::P*_14e_*-*dcaAKIJP* | genome integrated *dcaAKIJP* cluster under the control of P*_14e_* | this work  MiCat#623 |
| A1 | GRC3∆5-TYR2 *attTn7*::P*_14e_*-*dcaAKIJP* evolved on AA as sole carbon source | this work  MiCat#1323 |
| A2 | GRC3∆5-TYR2 *attTn7*::P*_14e_*-*dcaAKIJP* evolved on AA as sole carbon source | this work  MiCat#1324 |
| A3 | GRC3∆5-TYR2 *attTn7*::P*_14e_*-*dcaAKIJP* evolved on AA as sole carbon source | this work  MiCat#1325 |
| A4 | GRC3∆5-TYR2 *attTn7*::P*_14e_*-*dcaAKIJP* evolved on AA as sole carbon source | this work  MiCat#1326 |
| A5 | GRC3∆5-TYR2 *attTn7*::P*_14e_*-*dcaAKIJP* evolved on AA as sole carbon source | this work  MiCat#1327 |
| A6 | GRC3∆5-TYR2 *attTn7*::P*_14e_*-*dcaAKIJP* evolved on AA as sole carbon source | this work  MiCat#1328 |
| GRC3∆5-TYR2 Δ*psrA* | knockout of *psrA* | this work  MiCat#1426 |
| GRC3∆5-TYR2 *attTn7*::P*_14e_*-*dcaAKIJP* Δ*psrA* | knockout of *psrA* in addition to genomic integration of *dcaAKIJP* | this work  MiCat#1427 |
| A1 Δ*psrA* | knockout of *psrA* in evolved strain A1 | this work  MiCat#1428 |
| GRC3∆5-TYR2 AA | integration of *dcaAKIJP* at the position of *paaYX* by deleting *paaYX,* knockout of *psrA* | this work  MiCat#1434 |
| GRC3∆5-TYR2 *attTn7*::P*_14e_*-*dcaAKIJP* Δ*paaYX* Δ*psrA* | knockout of *paaYX* and *psrA* in addition to genomic integration of *dcaAKIJP* | this work  MiCat#1443 |
| GRC3∆5-TYR2 Δ*paaYX*:: P*_14e_*-*dcaAKIJP* | integration of *dcaAKIJP* at the position of *paaYX* by deleting of *paaYX* | this work  MiCat#1978 |
| GRC3∆5-TYR2 *attTn7*::P*_14e_*-*dcaAKIJP* Δ*paaYX* | knockout of *paaYX* in addition to genomic integration of *dcaAKIJP* | this work  MiCat#2399 |
| A1 *rpmE* (repaired) | repaired *rpmE* region with wild-type sequence | this work  MiCat#2233 |
| A2 *rpmE* (repaired) | repaired *rpmE* region with wild-type sequence | this work  MiCat#2234 |
| A3 *rpmE* (repaired) | repaired *rpmE* region with wild-type sequence | this work  MiCat#2235 |
| A5 *rpmE* (repaired) | repaired *rpmE* region with wild-type sequence | this work  MiCat#2244 |
| A6 *rpmE* (repaired) | repaired *rpmE* region with wild-type sequence | this work  MiCat#2245 |
| GRC3∆5-TYR2 BDO  *attTn7::nagR/PnagAa-rpcTAL* | combination of SNV in PVLB_10540, PVLB_12690 and PVLB_13305, *attTn7::Kan_FRT_nagR/PnagAa-rpcTAL* | this work  MiCat#2589 |
| GRC3∆5-TYR2 AA *attTn7::nagR/PnagAa-rpcTAL* | combined integration of *dcaAKIJP* at the position of *paaYX* by deleting *paaYX,* knockout of *psrA*, *attTn7::Kan_FRT_nagR/PnagAa-rpcTAL* | this work MiCat#2587 |

**Table S2:** Plasmids used in this work

| Plasmids | Description | Reference |
| --- | --- | --- |
| pRK2013 | Km^R^, *oriV*(ColE1) *mob*^+^ *tra*^+^ | Figurski and Helinski (1979) |
| pTNS1 | Amp^R^, *oriV*(R6K), *TnSABC*+*D* operon | Choi et al. (2005) |
| pBBFLP | helper plasmid used for antibiotic markers excision; *oriV*(pBBR1) *oriT* *mob^+^* λP_R_::FLP λ(cI857) *sacB tet*, Tc^R^ | de Las Heras et al. (2008) |
| pEMG | Km^R^, *oriV*(R6K), *lacZ*α with two flanking I-SceI sites | Martínez-García and de Lorenzo (2011) |
| pSNW2 | pSNW2, pEMG with *msfGFP* | Volke et al. (2020) |
| pSW-2 | Gm^R^, *oriV*(RK2), *xylS*, P_m_→Ι-sceΙ | Martínez-García and de Lorenzo (2011) |
| pBG14e_FRT_Kan_*dcaAKIJP* | pBG14e_FRT_Kan-derivative with *dcaAKIJP* from *A. baylyi* instead of *msfGFP* | Ackermann et al. (2021) |
| pSNW2_PVLB_10540^L480L^ | pSNW2 bearing the flanking regions of SNV in PVLB_10540 identified in *P. taiwanensis* AB1 | this work |
| pSNW2_PVLB_12690^A247V^ | pSNW2 bearing the flanking regions of SNV in PVLB_12690 identified in *P. taiwanensis* AB1 | this work |
| pSNW2_PVLB_13305^S141P^ | pSNW2 bearing the flanking regions of SNV in PVLB_13350 identified in *P. taiwanensis* AB1 | this work |
| pSNW2_PVLB_10765^G179D^ | pSNW2 bearing the flanking regions of SNV in PVLB_10765 identified in *P. taiwanensis* AB1 | this work |
| pSNW2_∆*psrA* | pSNW2 bearing the flanking regions of *psrA* | this work |
| pSNW2_*attTn7*_recycling | pSNW2 bearing the flanking regions of of any insert in *attTn7* site for its recycling | Schwanemann (2023) |
| pSNW2_∆*paaYX* | pSNW2 bearing the flanking regions of *paaYX* | this work |
| pSNW2_*∆paaYX*::*P_14e_-dcaAKIJP* | pSNW2 bearing the flanking regions of *paaYX* and the synthetic promoter 14e in combination with the *dcaAKIJP* from *A. baylyi* between these flanking regions | this work |
| pSNW2_cured*rpmE* | pSNW2 bearing the flanking regions of *rpmE* | this work |
| pBG_*Kan_FRT_*  *nagR/PnagAa-rpcTAL* | pBG14e_FRT_Kan-derivative with the salicylate-inducible *nagR/PnagAa* promoter system instead of the *P14e* and *rpcTAL* instead of *msfGFP* | this work |

**Table S3:** Oligonucleotides used in this work

| **Primer** | **Sequence 5´→3´** | **Template/purpose** |
| --- | --- | --- |
| LO01 | Tctagagtcgacctgcag | pSNW2 backbone |
| LO02 | Gaattcagattaccctgttatcc |  |
| LO32 | taacagggtaatctgaattcgcggcccagaacctgctg | genomic region around SNV in PVLB_10540^L480L^ |
| LO33 | gcctgcaggtcgactctagacctggatgatcttcgcggc |  |
| LO24 | taacagggtaatctgaattcgtacgggttggccgacga | genomic region around SNV in PVLB_12690^A247V^ |
| LO27 | gcctgcaggtcgactctagacttgacgtcggcctcgtcg |  |
| LO28 | taacagggtaatctgaattcgcatatcctgcccaacgc | genomic region around SNV in PVLB_13350^S141P^ |
| LO31 | gcctgcaggtcgactctagactcgaactgttcacaggcc |  |
| LO36 | taacagggtaatctgaattcctcgccaccggttcggcatag | genomic region around SNV in PVLB_10765^G179D^ |
| LO37 | gcctgcaggtcgactctagacgcgacccggccaaggcc |  |
| LO048 | cttcagtacgccaagaacg | qPCR PVLB_10545 |
| LO049 | gtgcgaacgccagctggtcatc |  |
| LO61 | ttccgacagttcgtgatcgcc | mapping of the deletion in PVLB_02465 and intergenic region between PVLB_ 02465 and *putA* |
| LO63 | aacagctacaccatcctgc |  |
| SK265 | taaaaaacgcaattggacgtcggcatcaaataaaac | pBELK for FRT-flanked Km^R^ |
| SK266 | acgtccaattgcgttttttattggtgag |  |
| SK267 | gttatggagcattttggtcatgagattatcg |  |
| SK268 | tgaccaaaatgctccataacatcaaacatc | pBG14b to pBG14g for promoter, BCD2, *msfGFP*, terminator T0 |
| SK269 | ttgtccaattatcagagattttgagacac |  |
| TL_344 | tgatcatgccgacagcatcg | qPCR *rpoD* |
| TL_345 | ttcagcgaatcgcgtacacg |  |


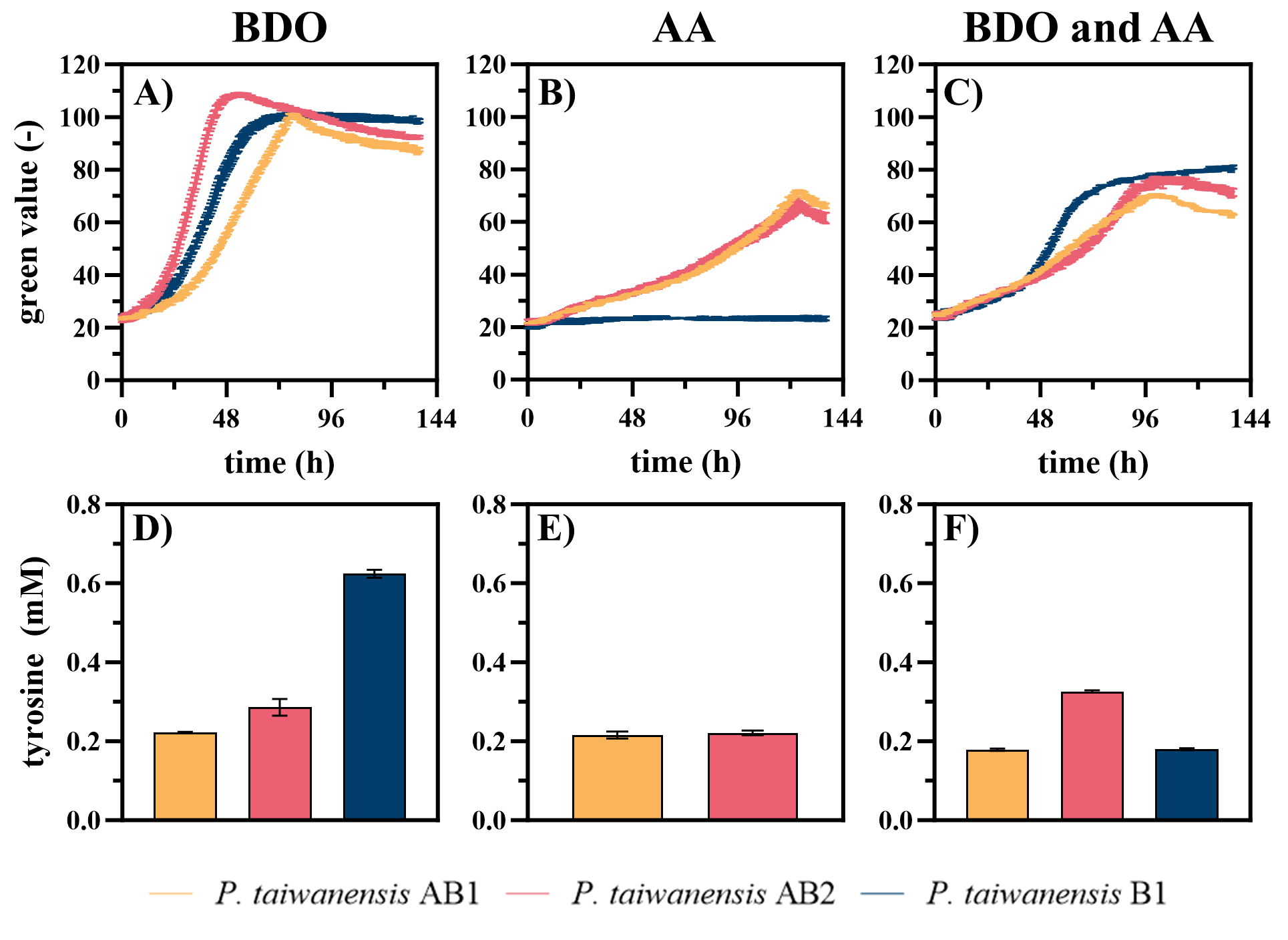


**Figure S1:** **Growth of P. taiwanensis strains evolved on BDO on BDO and AA and a mixture of both.** The cultivated strains were P. taiwanensis AB1 (yellow), AB2 (pink), AB1 (C) and B1 (blue). All strains were cultivated in three-fold buffered MSM with the indicated carbon sources at a total C-molar equivalent to 45 mM BDO. The strains were cultivated in in a Growth profiler with online monitoring of growth via green values. Error bars derive from three technological replicates and indicate the SEM.

**
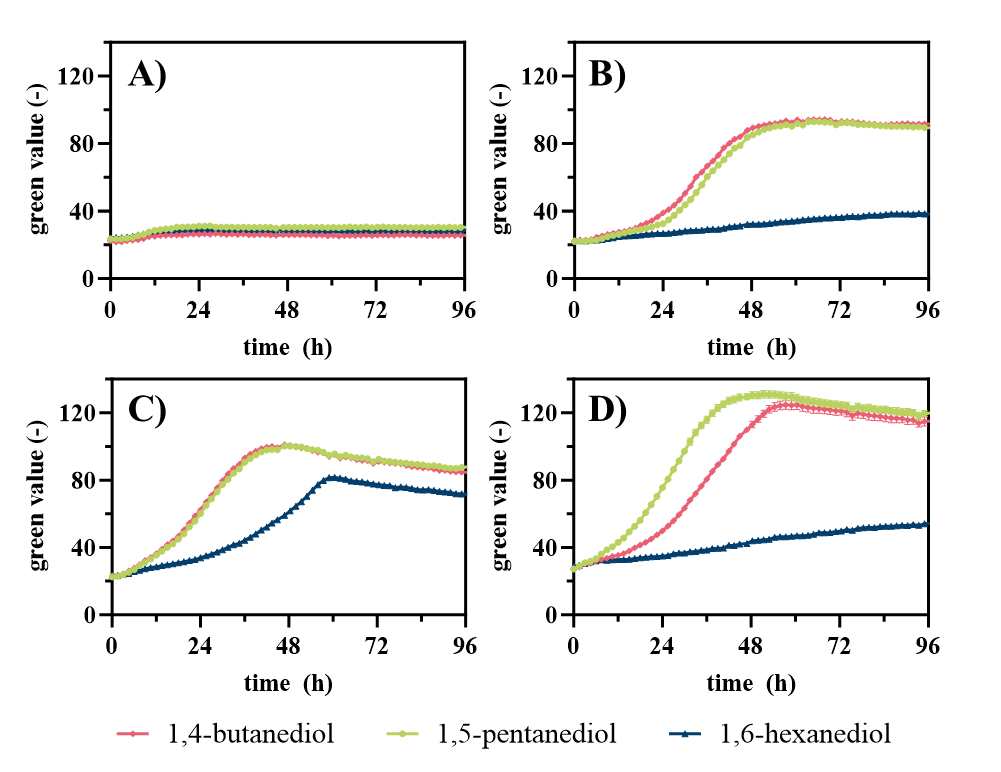
**

**Figure S2: Growth of P. taiwanensis strains evolved on BDO on diols with different chain lengths.** The cultivated strains were P. taiwanensis GRC3∆5-TYR2 (A), B1 (B), AB1 (C) and AB1 ΔattTn7::P_14e__dcaAKIJP (D). All strains were cultivated in three-fold buffered MSM with the indicated carbon sources at a C-molar equivalent to 45 mM BDO. The strains were cultivated in in a Growth profiler with online monitoring of growth via green values. Error bars derive from three technological replicates and indicate the SEM. In addition to BDO and AA, medium-chain-length (mcl) diols and dicarboxylates (DCA) are generally common products of the chemical and enzymatic hydrolysis of various polyesters and polyethers(Ackermann et al., 2024).To further expand the substrate spectra for the diols analogously to the dicarboxylic acids up to a chain length of six carbon atoms the growth of the evolved strains on 1,5-pentanediol and 1,6-hexanediol was tested. All strains were able to grow on 1,5-pentanediol. Interestingly, only the strain that had been evolved on AA was able to grow on 1,6-hexanediol, indicating that 1,6-hexanediol is metabolized via AA. This corresponds to one of two possible degradation pathways shown by Ackermann et al. (2024) for P. putida KT2440, in which the diol is completely oxidized to the dicarboxylic acid and subsequently CoA activated. Upon knockout of the dcaAKIJP, growth on 1,6-hexanediol was significantly reduced, which further promotes this hypothesis. However, minor growth was still detected indicating that an additional metabolic route contributing to 1,6-hexanediol assimilation is present, i.e., the degradation via partial oxidation to the hydroxy-acid followed by CoA activation (Ackermann et al., 2024).


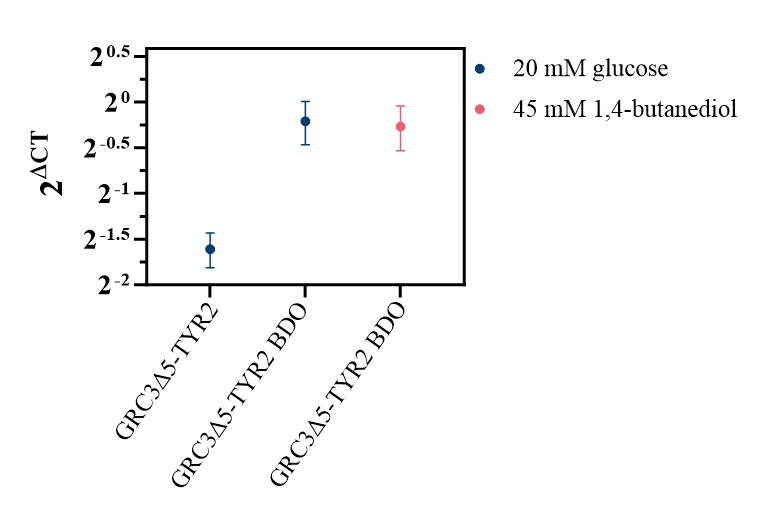


**Figure S3: RT-qPCR analysis of PVLB_10545 expression with and without the mutation PVLB_10540^L480L^.** P. taiwanensis GRC3∆5-TYR2 PVLB_10540^L480L^ PVLB_12690^A247V^ PVLB_13305^S141P^ was cultivated in MSM containing 20 mM glucose as well as MSM containing 45 mM BDO as sole carbon source and P. taiwanensis GRC3∆5-TYR2 was cultivated in MSM containing 20 mM glucose as sole carbon source. Samples were taken at an optical density of 0.9 to 1.1. The measured Ct values were normalized to expression of rpoD (Gene expression level = 2Ct(rpoD)-Ct(target)). Error bars derive from three biological replicates and indicate the SEM.


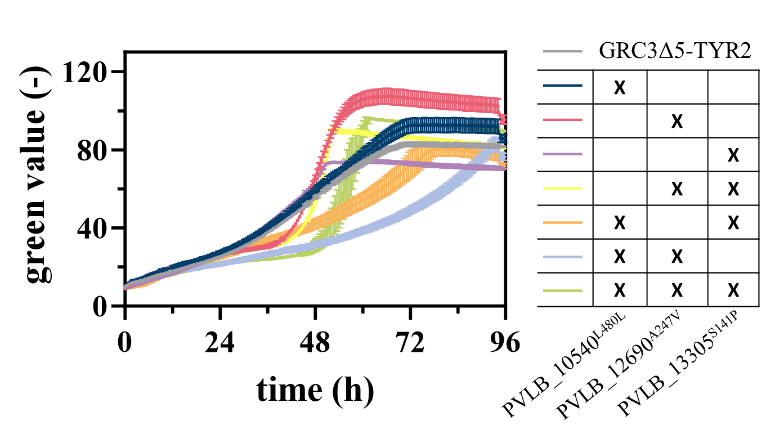


**Figure S4: Cultivation of reverse engineered P. taiwanensis GRC3Δ5-TYR2 strains for growth on 4-hydroxybutyrate**. Growth curves of engineered strains carrying different mutations found during the whole-genome sequencing of the ALE on BDO. All strains were cultivated in three-fold buffered MSM medium containing 45 mM of 4-hydroxybutyrate as sole carbon source in a Growth profiler with online monitoring of growth via green values. Error bars represent the SEM (n=3).


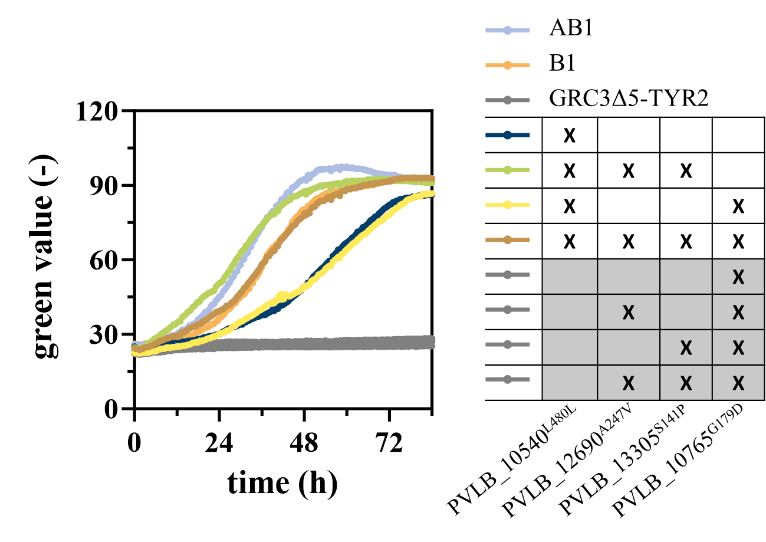


**Figure S5: Reverse engineering of P. taiwanensis GRC3Δ5-TYR2 strains for growth on BDO**. Growth curves of engineered strains carrying different mutations found during the whole-genome sequencing after the ALE on BDO. Non-growing strains are marked in the table by grey background color. All strains were cultivated in three-fold buffered MSM medium containing 45 mM of BDO as sole carbon source in a Growth profiler with online monitoring of growth via green values. Error bars represent the SEM (n=3).

**Figure S6: Growth of the evolved and the corresponding repaired *rpmE* strains with different zinc concentrations.** The following evolved and corresponding repaired *rpme* strains were cultivated: A1 (A); A2 (B); A3 (C).All strains were cultivated in three-fold buffered MSM containing 30 mM AA and different zinc concentration. Growth curves were measured in a Growth profiler with online monitoring of growth via green values. Shaded areas indicate the SEM, but they are not visible as they are too small and therefore overlaid by the lines indicating the mean (n=3). The *rpmE* gene encodes the zinc-dependent (C+) ribosomal protein bL31. No Zn-independent (C–) paralogs were identified in *P. taiwanensis* in contrast to other bacteria such a*s P. aeruginosa* or *E. coli* (Hensley et al., 2012). In those, the presence of C– paralogs is linked to zinc availability, enabling adaptation to zinc fluctuations (Hensley et al., 2012). Increased Zn²⁺ levels improved growth of strains harboring the wild-type *rpmE*, whereas strains with a mutated *rpmE* displayed reduced sensitivity to zinc limitation. Nonetheless, even high zinc supplementation did not restore growth to the level of ALE strains, suggesting that zinc availability alone does not explain the observed growth differences.


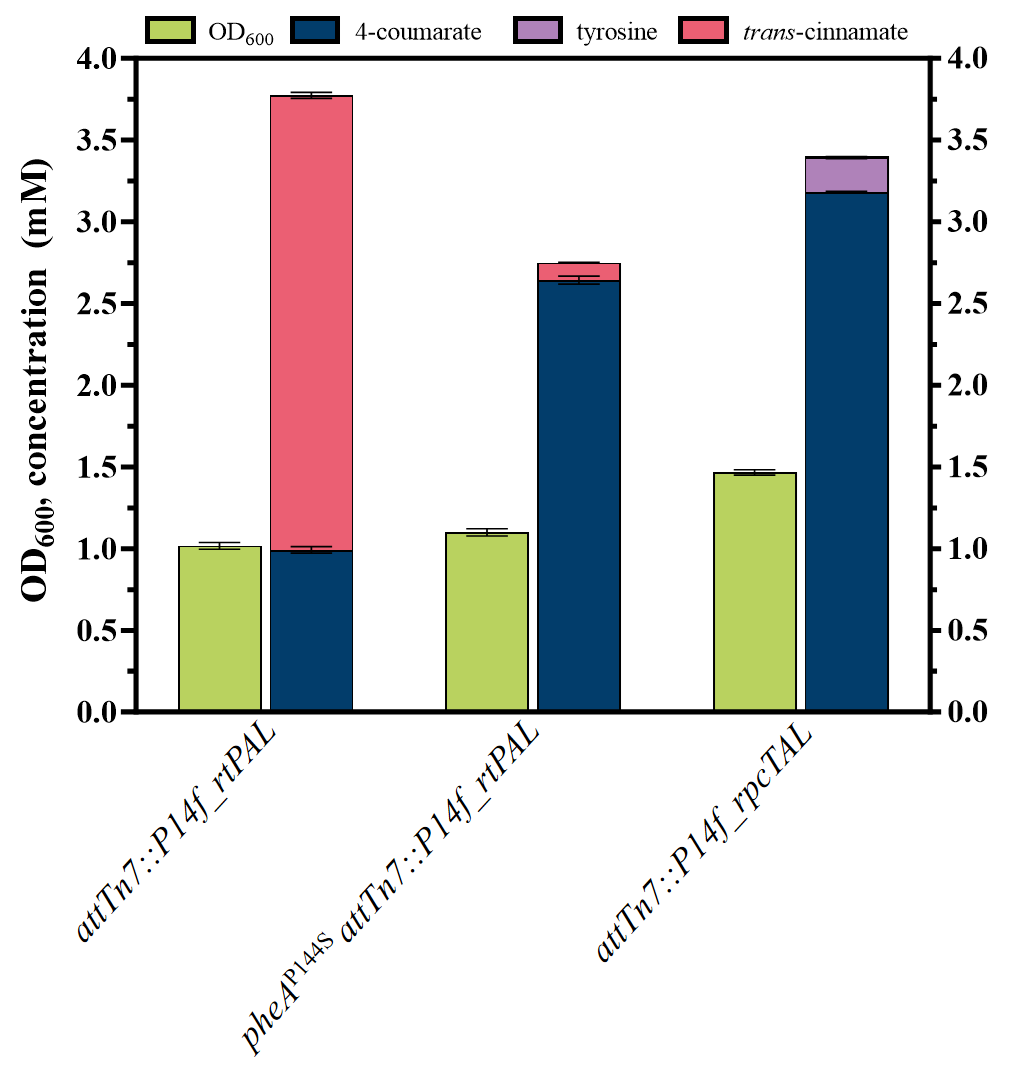


**Figure S7: Comparison of 4-coumarate production with the *Rt*PAL and the *Rpc*TAL.** All strains were cultivated in 1.5 mL two-fold buffered MSM medium containing 20 mM glucose within System Duetz. Samples were taken after 96 hours and the OD_600_ as well as concentrations of 4-coumarate, *trans*-cinnamate and tyrosine were measured. The concentrations of all measured compounds are added in the bar chars. Error bars represent the SEM and derive from six technical replicates for both strains harboring *Rt*PAL and three technical replicates for the strain harboring the *Rpc*TAL.


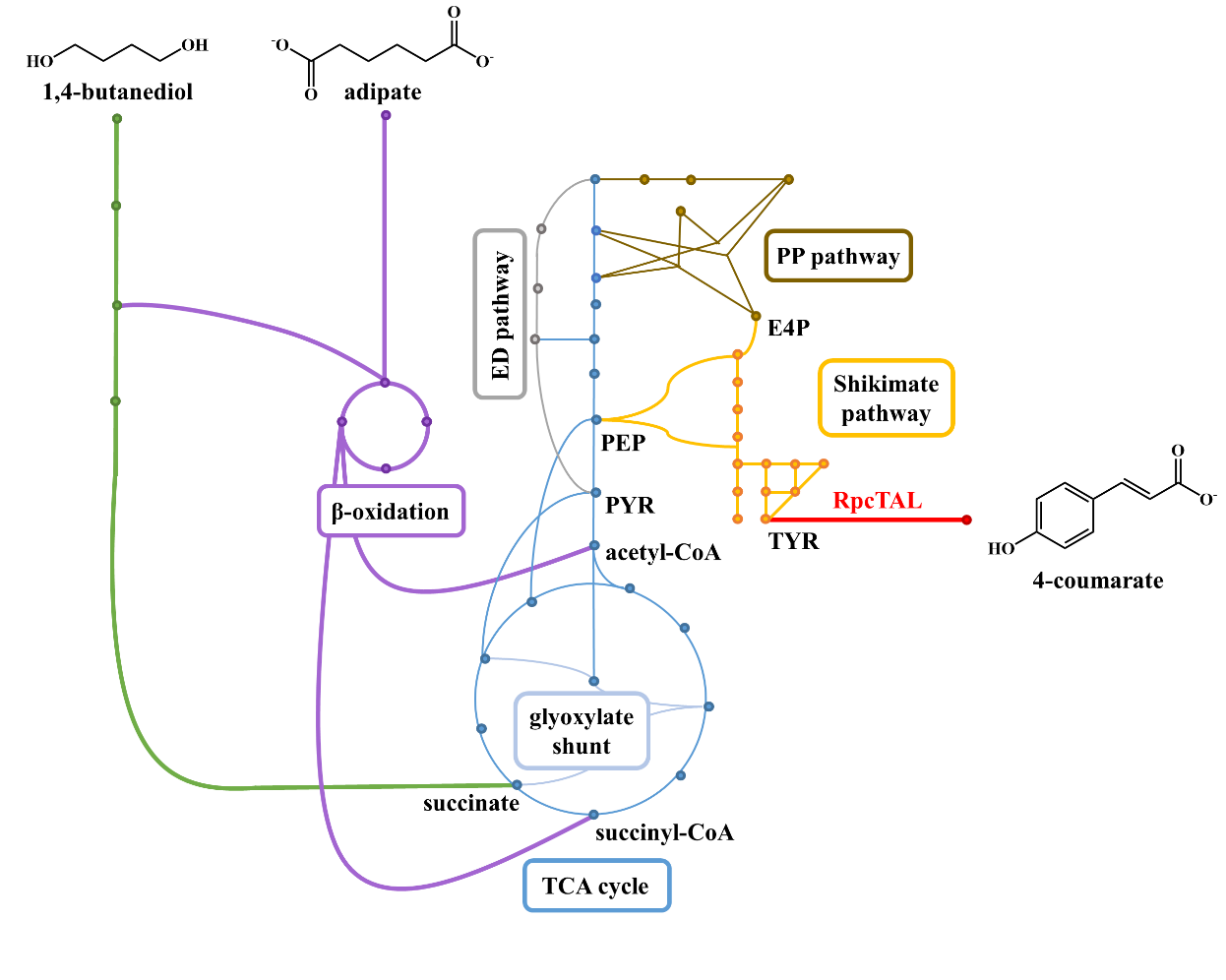


**Figure S8: Schematic representation of the central native and engineered metabolic pathways leading to 4-coumarate production in Pseudomonas taiwanensis from BDO and AA as substrates.** The catabolism of BDO proceeds through direct oxidation to succinate (green) or β-oxidation (purple) and AA through β-oxidation (purple), feeding into central metabolism via succinate, succinyl-CoA and acetyl-CoA. These intermediates enter the central carbon metabolism via the tricarboxylic acid (TCA) cycle (blue). The carbon flux is then directed via gluconeogenic reactions and the pentose phosphate pathway (PP pathway, brown) toward phosphoenolpyruvate (PEP) and erythrose-4-phosphate (E4P), key precursors for the shikimate pathway (yellow). The shikimate pathway leads to the biosynthesis of L-tyrosine (TYR), which is subsequently converted to 4-coumarate by the heterologously expressed RpcTAL (red).

**Supporting information 1: Mutations in regulatory region of the *paa* genecluster:**

Deletions are marked in red. Insertions are marked in yellow and SNVs are marked in green. Putative promoter of *paaF* is underlined.

| ALE A1 |
| --- |
| *paaYX:* |
| atgagcaaccttgccccactcaatactctgatcactcgctttcaggaacagacgccgattcgcgccagttcgctgatcattaccctgtacggcgatgccatcgagccccacggcggcaccgtgtggctgggcagcttgatcaacctgctggagccgatcggcatcaacgagaggttgattcgcacgtcgatctttcgcctgaccaaagagggatggctcacggccgaaaaggttggccgacgcagctattacagtttgaccggcaccggccgccgacgcttcgagaaagctttcaagcgggtctatagtccaagccagccagcgtgggacggtgcctggacactggtgctcctgtcgcagctggaagcggtcaagcgcaaaaacgtgcgcgaagagttggagtggcaaggctttggcgccatcgcccccaacgtgttgggctgcccccgtgccgaccgtaccgacctcgccgcgaccttgcgcgagctcgatgccgacgaggacagcatcgtcttcgaaacccatgcccaggaagtgctggcctccaaggccatgcgcgcgcaagttcgcgagagctggcgcatcgatgaactgggtcagcactacagtgaattcatccgcttgttcaggccgttgtggcagggactgaaggagcaggaacaactggacgcccaggactgcttcctggcgcgcaccctgttgatccacgagtaccgtcgtttgctgctgcgcgacccgcaattaccagacgagctgttgccaggggactgggagggcagggccgcacgccagttgtgccgcaacctgtatcgtttggtatttgccaaggctgaagaatggctaaacagcgcactggagaccgcagacgggccgttgccagaggtgggggagagtttctacaagcgttttggtggcttggtgtag |

| ALE A2 |
| --- |
| *paaYX:* |
| atgagcaaccttgccccactcaatactctgatcactcgctttcaggaacagacgccgattcgcgccagttcgctgatcattaccctgtacggcgatgccatcgagccccacggcggcaccgtgtggctgggcagcttgatcaacctgctggagccgatcggcatcaacgagaggttgattcgcacgtcgatctttcgcctgaccaaagagggatggctcacggccgaaaaggttggccgacgcagctattacagtttgaccggcaccggccgccgacgcttcgagaaagctttcaagcgggtctatagtccaagccagccagcgtgggacggtgcctggacactggtgctcctgtcgcagctggaagcggtcaagcgcaaaaacgtgcgcgaagagttggagtggcaaggctttggcgccatcgcccccaacgtgttgggctgcccccgtgccgaccgtaccgacctcgccgcgaccttgcgcgagctcgatgccgacgaggacagcatcgtcttcgaaacccatgcccaggaagtgctggcctccaaggccatgcgcgcgcaagttcgcgagagctggcgcatcgatgaactgggtcagcactacagtgaattcatccgcttgt[Insertion 99 bp] tcaggccgttgtggcagggactgaaggagcaggaacaactggacgcccaggactgcttcctggcgcgcaccctgttgatccacgagtaccgtcgtttgctgctgcgcgacccgcaattaccagacgagctgttgccaggggactgggagggcagggccgcacgccagttgtgccgcaacctgtatcgtttggtatttgccaaggctgaagaatggctaaacagcgcactggagaccgcagacgggccgttgccagaggtgggggagagtttctacaagcgttttggtggcttggtgtag |

| ALE A3 |
| --- |
| *paaF* IGR *paaY* |
| gtcgtattcctcaggctgctcgcgcggcgcagtggatggccggcttttattggaattgtttgcaggggcgcggcacgaaccacgctcaggtatttcagtatatgccttatgcgatacacaatatcaagcatgaatacgtgattat[g>a]tatccaatagtgcgcgtgatgcattgatggaaccgcggtttgacctgacgaaatcccgcagaaaatggttgaaaggcgatttctggcgggcgctaatcacgcgcatccacgctgaaaaaagcgatacatggatgatggcaattgacgaaaaatatcgaagtgatacaagatcaagcaacgcttcaaatcatttctggaggtgcgag |

| ALE A4 |
| --- |
| *paaF* IGR *paaY* |
| gtcgtattcctcaggctgctcgcgcggcgcagtggatggccggcttttattggaattgtttgcaggggcgcggcacgaaccacgctcaggtatttcagtatatgccttatgcgatacacaatatcaagcatgaatacgtgattatgt[a>t]tccaatagtgcgcgtgatgcattgatggaaccgcggtttgacctgacgaaatcccgcagaaaatggttgaaaggcgatttctggcgggcgctaatcacgcgcatccacgctgaaaaaagcgatacatggatgatggcaattgacgaaaaatatcgaagtgatacaagatcaagcaacgcttcaaatcatttctggaggtgcgag |

| ALE A5 |
| --- |
| *paaYX:* |
| Atgagcaaccttgccccactcaatactctgatcactcgctttcaggaacagacgccgattcgcgccagttcgctgatcattaccctgtacggcgatgccatcgagccccacggcggcaccgtgtggctgggcagcttgatcaacctgctggagccgatcggcatcaacgagaggttgattcgcacgtcgatctttcgcctgaccaaagagggatggctcacggccgaaaaggttggccgacgcagctattacagtttgaccggcaccggccgccgacgcttcgagaaagctttcaagcgggtctatagtccaagccagccagcgtgggacggtgcctggacactggtgctcctgtcgcagctggaagcggtcaagcgcaaaaacgtgcgcgaagagttggagtggcaaggctttggcgccatcgcccccaacgtgttgggctgcccccgtgccgaccgtaccgacctcgccgcgaccttgcgcgagctcgatgccgacgaggacagcatcgtcttcgaaacccatgcccaggaagtgctggcctccaaggccatgcgcgcgcaagttcgcgagagctggcgcatcgatgaactgggtcagcactacagtgaattcatccgcttgttcaggccgttgtggcagggactgaaggagcaggaacaactggacgcccaggactgcttcctggcgcgcaccctgttgatccacgagtac[c>a]gtcgtttgctgctgcgcgacccgcaattaccagacgagctgttgccaggggactgggagggcagggccgcacgccagttgtgccgcaacctgtatcgtttggtatttgccaaggctgaagaatggctaaacagcgcactggagaccgcagacgggccgttgccagaggtgggggagagtttctacaagcgttttggtggcttggtgtag |

| ALE A6 |
| --- |
| *paaF* IGR *paaY* |
| gtcgtattcctcaggctgctcgcgcggcgcagtggatggccggcttttattggaattgtttgcaggggcgcggcacgaaccacgctcaggtatttcagtatatgccttatgcgatacacaatatcaagcatgaatacgtgattatg[t>c]atccaatagtgcgcgtgatgcattgatggaaccgcggtttgacctgacgaaatcccgcagaaaatggttgaaaggcgatttctggcgggcgctaatcacgcgcatccacgctgaaaaaagcgatacatggatgatggcaattgacgaaaaatatcgaagtgatacaagatcaagcaacgcttcaaatcatttctggaggtgcgag |

**Supporting information 2: Mutations occurred around *rpmE*:**

Deletions are marked in red. SNVs are marked in green. In blue multiple breakpoint, not further investigated Putative promotersite of *rpmE* is underlined.

| ALE A1 |
| --- |
| IGR |
| gcggaaggtctcggcagggtatttcaacgagtggcgagactagcagacgaccgtcggtgcaagcgacaacttgcgctgacgctgtgctctggtattattcgccgcctaattacgtgcggtattcaacaattggtgttgggtggcggcacgcagctcgaggaagtgacc |
| *rpmE* |
| atgaaagcagatattcatccgaactacgaagtagttgcagtcacctgcagctgcggcaacaaattcgaaacccgttcgaccctggccaagccgctggcgatcgacgtgtgctcccagtgccacccgttctacactggtaagcagaaagtcctggacaccggtggtcgcgtacagaagttcgccgatcgcttcggtatgttcggtaccaagaagtaa |
| IGR |
| tc |
| nuclease |
| atgcgcatggcgaatccttcgggcttcgcattgctgcaaaaaaaggcgccccttgtgggcgctttttttgtgggcgcgatgtggcattttccggctctggcgttctgcccgctgccggaaaacccgcagatggtagccgtgcgacagatcgtcgatggcgataccttgcgcctgaccgacggtcgcagtgtgcgtctgatcggtatcaatgccccggagattgggcgccaagggcgcagcagtgagccttacgccgaaatggccaggcaacgcctgcaggcgttggtcaaggccagtgatggccgcgtaggcctggtgccgggtgtcgaggcaaaggacaggtacggccgcaccttggcccatgtctacggccgcaatggcgacaatttcgaagctcgtttactcagcgaggggcttggctatcgtgtcgcggtcgcgcccaatgttcgcctcgccggttgccagcagagcgccgaacaggcagcgcgtgcttccggagcggggttgtggcgacgttcaccggtggtgcgcggtggcgatgtcaaacggtccgggtttgccgtgatcggcggcagaatcaccggcatcgagcgcaatcgcggtggtgtctggctcgaactcgacgatgcgctggtgctgcaggttcccgctcgtctgcaacgcaacttcccttcgagcttcttcgataacctcaagggacgccaggtcgaagcgcgtggctgggtgctggaccgttcccgcaagggggggcttaagccagggcagcgacgctgggtgttgccattgactgatccgagcatgctggagcgcgtttcacggtga |

| ALE A2 |
| --- |
| IGR |
| gcggaaggtctcggcagggtatttcaacgagtggcgagactagcagacgaccgtcggtgcaagcgacaacttgcgctgacgctgtgctctggtattattcgccgcctaattacgtgcggtattcaacaattggtgttgggtggcggcacgcagctcgaggaagtgacc |
| *rpmE* |
| Atgaaagcagatattcatccgaactacgaagtagttgcagtcacctgcagctgcggcaacaaattcgaaacccgttcgaccctggccaagccgctggcgatcgacgtg[t>a]gctcccagtgccacccgttctacactggtaagcagaaagtcctggacaccggtggtcgcgtacagaagttcgccgatcgcttcggtatgttcggtaccaagaagtaa |
| IGR |
| tc |
| nuclease |
| atgcgcatggcgaatccttcgggcttcgcattgctgcaaaaaaaggcgccccttgtgggcgctttttttgtgggcgcgatgtggcattttccggctctggcgttctgcccgctgccggaaaacccgcagatggtagccgtgcgacagatcgtcgatggcgataccttgcgcctgaccgacggtcgcagtgtgcgtctgatcggtatcaatgccccggagattgggcgccaagggcgcagcagtgagccttacgccgaaatggccaggcaacgcctgcaggcgttggtcaaggccagtgatggccgcgtaggcctggtgccgggtgtcgaggcaaaggacaggtacggccgcaccttggcccatgtctacggccgcaatggcgacaatttcgaagctcgtttactcagcgaggggcttggctatcgtgtcgcggtcgcgcccaatgttcgcctcgccggttgccagcagagcgccgaacaggcagcgcgtgcttccggagcggggttgtggcgacgttcaccggtggtgcgcggtggcgatgtcaaacggtccgggtttgccgtgatcggcggcagaatcaccggcatcgagcgcaatcgcggtggtgtctggctcgaactcgacgatgcgctggtgctgcaggttcccgctcgtctgcaacgcaacttcccttcgagcttcttcgataacctcaagggacgccaggtcgaagcgcgtggctgggtgctggaccgttcccgcaagggggggcttaagccagggcagcgacgctgggtgttgccattgactgatccgagcatgctggagcgcgtttcacggtga |

| ALE A3 |
| --- |
| IGR |
| gcggaaggtctcggcagggtatttcaacgagtggcgagactagcagacgaccgtcggtgcaagcgacaacttgcgctgacgctgtgctctggtatta[t>c]tcgccgcctaattacgtgcggtattcaacaattggtgttgggtggcggcacgcagctcgaggaagtgacc |
| *rpmE* |
| atgaaagcagatattcatccgaactacgaagtagttgcagtcacctgcagctgcggcaacaaattcgaaacccgttcgaccctggccaagccgctggcgatcgacgtgtgctcccagtgccacccgttctacactggtaagcagaaagtcctggacaccggtggtcgcgtacagaagttcgccgatcgcttcggtatgttcggtaccaagaagtaa |
| IGR |
| tc |
| nuclease |
| atgcgcatggcgaatccttcgggcttcgcattgctgcaaaaaaaggcgccccttgtgggcgctttttttgtgggcgcgatgtggcattttccggctctggcgttctgcccgctgccggaaaacccgcagatggtagccgtgcgacagatcgtcgatggcgataccttgcgcctgaccgacggtcgcagtgtgcgtctgatcggtatcaatgccccggagattgggcgccaagggcgcagcagtgagccttacgccgaaatggccaggcaacgcctgcaggcgttggtcaaggccagtgatggccgcgtaggcctggtgccgggtgtcgaggcaaaggacaggtacggccgcaccttggcccatgtctacggccgcaatggcgacaatttcgaagctcgtttactcagcgaggggcttggctatcgtgtcgcggtcgcgcccaatgttcgcctcgccggttgccagcagagcgccgaacaggcagcgcgtgcttccggagcggggttgtggcgacgttcaccggtggtgcgcggtggcgatgtcaaacggtccgggtttgccgtgatcggcggcagaatcaccggcatcgagcgcaatcgcggtggtgtctggctcgaactcgacgatgcgctggtgctgcaggttcccgctcgtctgcaacgcaacttcccttcgagcttcttcgataacctcaagggacgccaggtcgaagcgcgtggctgggtgctggaccgttcccgcaagggggggcttaagccagggcagcgacgctgggtgttgccattgactgatccgagcatgctggagcgcgtttcacggtga |

| ALE A4 |
| --- |
| IGR |
| gcggaaggtctcggcagggtatttcaacgagtggcgagactagcagacgaccgtcggtgcaagcgacaacttgcgctgacgctgtgctctggtattattcgccgcctaattacgtgcggtattcaacaattggtgttgggtggcggcacgcagctcgaggaagtgacc |
| *rpmE* |
| atgaaagcagatattcatccgaactacgaagtagttgcagtcacctgcagctgcggcaacaaattcgaaacccgttcgaccctggccaagccgctggcgatcgacgtgtgctcccagtgccacccgttctacactggtaagcagaaagtcctggacaccggtggtcgcgtacagaagttcgccgatcgcttcggtatgttcggtaccaagaagtaa |
| IGR |
| tc |
| nuclease |
| atgcgcatggcgaatccttcgggcttcgcattgctgcaaaaaaaggcgccccttgtgggcgctttttttgtgggcgcgatgtggcattttccggctctggcgttctgcccgctgccggaaaacccgcagatggtagccgtgcgacagatcgtcgatggcgataccttgcgcctgaccgacggtcgcagtgtgcgtctgatcggtatcaatgccccggagattgggcgccaagggcgcagcagtgagccttacgccgaaatggccaggcaacgcctgcaggcgttggtcaaggccagtgatggccgcgtaggcctggtgccgggtgtcgaggcaaaggacaggtacggccgcaccttggcccatgtctacggccgcaatggcgacaatttcgaagctcgtttactcagcgaggggcttggctatcgtgtcgcggtcgcgcccaatgttcgcctcgccggttgccagcagagcgccgaacaggcagcgcgtgcttccggagcggggttgtggcgacgttcaccggtggtgcgcggtggcgatgtcaaacggtccgggtttgccgtgatcggcggcagaatcaccggcatcgagcgcaatcgcggtggtgtctggctcgaactcgacgatgcgctggtgctgcaggttcccgctcgtctgcaacgcaacttcccttcgagcttcttcgataacctcaagggacgccaggtcgaagcgcgtggctgggtgctggaccgttcccgcaagggggggcttaagccagggcagcgacgctgggtgttgccattgactgatccgagcatgctggagcgcgtttcacggtga |

| ALE A5 |
| --- |
| IGR |
| Gcggaaggtctcggcagggtatttcaacgagtggcgagactagcagacgaccgtcggtgcaagcgacaacttgcgctgacgctgtgctctggt[a>g]ttattcgccgcctaattacgtgcggtattcaacaattggtgttgggtggcggcacgcagctcgaggaagtgacc |
| *rpmE* |
| Atgaaagcagatattcatccgaactacgaagtagttgcagtcacctgcagctgcggcaacaaattcgaaacccgttcgaccctggccaagccgctggcgatcgacgtgtgctcccagtgccacccgttctacactggtaagcagaaagtcctggacaccggtggtcgcgtacagaagttcgccgatcgcttcggtatgttcggtaccaagaagtaa |
| IGR |
| tc |
| nuclease |
| atgcgcatggcgaatccttcgggcttcgcattgctgcaaaaaaaggcgccccttgtgggcgctttttttgtgggcgcgatgtggcattttccggctctggcgttctgcccgctgccggaaaacccgcagatggtagccgtgcgacagatcgtcgatggcgataccttgcgcctgaccgacggtcgcagtgtgcgtctgatcggtatcaatgccccggagattgggcgccaagggcgcagcagtgagccttacgccgaaatggccaggcaacgcctgcaggcgttggtcaaggccagtgatggccgcgtaggcctggtgccgggtgtcgaggcaaaggacaggtacggccgcaccttggcccatgtctacggccgcaatggcgacaatttcgaagctcgtttactcagcgaggggcttggctatcgtgtcgcggtcgcgcccaatgttcgcctcgccggttgccagcagagcgccgaacaggcagcgcgtgcttccggagcggggttgtggcgacgttcaccggtggtgcgcggtggcgatgtcaaacggtccgggtttgccgtgatcggcggcagaatcaccggcatcgagcgcaatcgcggtggtgtctggctcgaactcgacgatgcgctggtgctgcaggttcccgctcgtctgcaacgcaacttcccttcgagcttcttcgataacctcaagggacgccaggtcgaagcgcgtggctgggtgctggaccgttcccgcaagggggggcttaagccagggcagcgacgctgggtgttgccattgactgatccgagcatgctggagcgcgtttcacggtga |

| ALE A6 |
| --- |
| IGR |
| Gcggaaggtctcggcagggtatttcaacgagtggcgagactagcagacgaccgtcggtgcaagcgacaacttgcgctgacgctgtgctctggtattattcgccgcctaattacgtgcggtattcaacaattggtgttgggtggcggcacgcagctcgaggaagtgacc |
| *rpmE* |
| Atgaaagcagatattcatccgaactacgaagtagttgcagtcacctgcagctgcggcaacaaattcgaaacccgttcgaccctggccaagccgctggcgatcgacgtgtgctcccagtgccacccgttctacactggtaagcagaaagtcctggacaccggtggtcgcgtacagaagttcgccgatcgcttcggtatgttcggtaccaagaagtaa |
| IGR |
| tc |
| nuclease |
| atgcgcatggcgaatccttcgggcttcgcattgctgcaaaaaaaggcgccccttgtgggcgctttttttgtgggcgcgatgtggcattttccggctctggcgttctgcccgctgccggaaaacccgcagatggtagccgtgcgacagatcgtcgatggcgataccttgcgcctgaccgacggtcgcagtgtgcgtctgatcggtatcaatgccccggagattgggcgccaagggcgcagcagtgagccttacgccgaaatggccaggcaacgcctgcaggcgttggtcaaggccagtgatggccgcgtaggcctggtgccgggtgtcgaggcaaaggacaggtacggccgcaccttggcccatgtctacggccgcaatggcgacaatttcgaagctcgtttactcagcgaggggcttggctatcgtgtcgcggtcgcgcccaatgttcgcctcgccggttgccagcagagcgccgaacaggcagcgcgtgcttccggagcggggttgtggcgacgttcaccggtggtgcgcggtggcgatgtcaaacggtccgggtttgccgtgatcggcggcagaatcaccggcatcgagcgcaatcgcggtggtgtctggctcgaactcgacgatgcgctggtgctgcaggttcccgctcgtctgcaacgcaacttcccttcgagcttcttcgataacctcaagggacgccaggtcgaagcgcgtggctgggtgctggaccgttcccgcaagggggggcttaagccagggcagcgacgctgggtgttgccattgactgatccgagcatgctggagcgcgtttcacggtga |
